# Adolescent Binge Ethanol Exposure Confers Lasting Adult Alcohol Tolerance due to Neuroimmune Activation: Reversal by Inhibition of HMGB1

**DOI:** 10.1111/adb.70119

**Published:** 2026-03-04

**Authors:** Fulton T. Crews, Ryan P. Vetreno

**Affiliations:** ^1^ Bowles Center for Alcohol Studies, School of Medicine University of North Carolina at Chapel Hill Chapel Hill North Carolina USA; ^2^ Department of Psychiatry, School of Medicine University of North Carolina at Chapel Hill Chapel Hill North Carolina USA; ^3^ Department of Pharmacology, School of Medicine University of North Carolina at Chapel Hill Chapel Hill North Carolina USA

**Keywords:** acute alcohol response, adolescent binge drinking, neuroinflammation, tolerance

## Abstract

Epidemiological studies suggest heavy adolescent binge drinking is strongly associated with later development of an alcohol use disorder (AUD). Alcohol tolerance (i.e., an acquired reduction in acute alcohol responsivity) is a universally recognized symptom of AUD, but the direct contribution of adolescent binge drinking to adult alcohol tolerance is poorly understood. To investigate the contributions of adolescent binge ethanol exposure to lasting acquisition of acute tolerance, we used our ethanol response battery (ERB) to assess intoxication rating, hypothermia, motor coordination and balance across cumulative ethanol doses (i.e., 0.0, 0.5, 1.0, 2.0 and 3.0 g/kg) in adult female Wistar rats following adolescent intermittent ethanol (AIE), lipopolysaccharide (LPS) and glycyrrhizic acid treatment following AIE. We report AIE confers lasting alcohol tolerance across cumulative ethanol doses and blunts acute ethanol‐induced increases in proinflammatory HMGB1 plasma levels. Adolescent LPS (1.0 mg/kg, i.p.) treatment, which mimics AIE‐induced HMGB1‐mediated neuroinflammation, induces adult alcohol tolerance and blunts HMGB1 release across cumulative ethanol doses on the ERB. Assessment of proinflammatory HMGB1 involvement in AIE‐induced acquisition of lasting alcohol tolerance showed that post‐AIE administration of the HMGB1 inhibitor glycyrrhizic acid reversed the AIE‐induced acquisition of alcohol tolerance in adulthood. These data reveal that (1) adolescent binge drinking confers long‐lasting low ethanol responsivity (i.e., tolerance), (2) proinflammatory neuroimmune activation contributes to the development of alcohol tolerance and (3) blockade of proinflammatory HMGB1 signalling reverses AIE‐induced acquisition of alcohol tolerance in adulthood. These findings suggest a potential mechanistic target for the development of novel therapeutics for the treatment of AUD.

## Graphical abstract

Adolescent intermittent ethanol (AIE) exposure induces enduring adult alcohol tolerance across a cumulative ethanol response battery. Parallel adolescent lipopolysaccharide challenge recapitulated tolerance and blunted acute ethanol–evoked HMGB1 release, implicating neuroimmune programming. Post‐AIE administration of the HMGB1 inhibitor glycyrrhizic acid normalized behavioural and physiological responsivity, and reduced HMGB1/RAGE/NF‐κB indices, identifying HMGB1 signalling as a reversible mechanistic driver of tolerance and a therapeutic target for AUD.

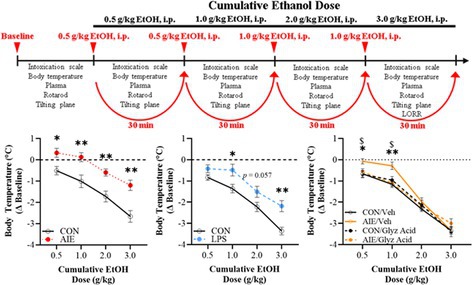

## 1 Introduction

Alcohol tolerance is a commonly endorsed symptom of alcohol use disorder (AUD) [1, 2] that likely contributes to increased binge drinking and risk of alcohol dependence. A low level of alcohol responsivity (i.e., tolerance) predicts later development of binge drinking and increased risk for lifelong alcohol problems [3]. Epidemiological studies report that an adolescent age of drinking onset is a significant risk factor for later development of an AUD [4, 5, 6, 7, 8, 9]. Binge drinking is common during adolescence, which involves rapid consumption of multiple drinks (4–5 drinks over a 2‐h period), contributing to the development of AUD [10]. Preclinical [11] and clinical studies [12] find adolescent drinking impacts brain development, which also increases risks for AUD. In this study, we systematically investigated the impact of adolescent intermittent ethanol (AIE), a preclinical rodent model of human adolescent binge drinking, on adult behavioural and physiological alcohol responsivity across multiple measures during an acute cumulative ethanol challenge [13].

Assessment of alcohol responsivity is affected by metabolism, time after ethanol and rising or falling blood ethanol levels (BECs). One consistent finding is that, compared to adults, adolescents have a low response to alcohol [14]. Using the tilting plane, White and colleagues [15] reported that acute ethanol elicited low responsivity in adolescent rats (postnatal day [P]30–P65) relative to adults (P70–P105), with adolescents developing chronic tolerance, but not adults with identical ethanol exposure. Similarly, chronic intermittent ethanol treatment of male rats during adolescence altered loss of righting reflex (LORR) sleep time, but not the aerial righting reflex, in adulthood [16]. However, adolescent alcohol responsivity is endpoint‐specific as adolescents are relatively less sensitive than adults to many aversive/sedative effects (e.g., motor impairment, sedation) yet often exhibit greater sensitivity to ethanol's rewarding and social facilitatory effects [17, 18, 19]. We recently compared adolescent (P40) and adult (P85) male and female rat ethanol responsivity using an ethanol response battery (ERB) cumulative dose response that assesses intoxication, hypothermia, rotarod balance, tilting plane motor coordination and LORR. Across cumulative ethanol doses and endpoints, adolescent males and females were significantly less sensitive to ethanol than adults [14]. While adolescents often display reduced sensitivity to several intoxicating/aversive alcohol effects, developmental exposure can also shift reactivity in the opposite direction. Notably, repeated adolescent ethanol has been shown to sensitize social‐anxiolytic responses whereas prenatal ethanol exposure can heighten ethanol appetitive reinforcement, highlighting endpoint‐specific plasticity that complements our central hypothesis about intermittent exposure and tolerance [18, 20]. Although the mechanisms underlying this age‐associated differential response to alcohol are unknown, the ERB provides a full ethanol dose response curve, allowing determination of whether AIE confers alcohol tolerance in adulthood.

Prior AIE studies found increased adult alcohol drinking and preference, increased anxiety and pain sensitivity, and cognitive deficits, all of which could increase risk for development of AUD [11]. Studies of adult acute ethanol induction of immediate early genes following AIE found reduced cFos + IR in prefrontal cortex across multiple doses, consistent with tolerance [21]. However, tolerance to alcohol is poorly understood and linked to multiple neurobiological systems [1, 13]. Chronic ethanol exposure studies support excitatory and inhibitory synaptic changes as contributing to the development of physical dependence, and the hyperexcitability of alcohol withdrawal [22] has been proposed to contribute to alcohol‐induced tolerance. Although our AIE model does not induce physical dependence, it does alter adult behaviours that increase risks for AUD development [11]. Previous studies find AIE induces long‐lasting changes in brain gene expression through epigenetic mechanisms that persist long after the last ethanol exposure [23]. We also find AIE increases adult expression of multiple proinflammatory cytokines including HMGB1, which is a nuclear cytokine known to be released by acute ethanol and activate epigenetic changes in neurons [24], proinflammatory activation of glia [25], and persistently alter behaviour [26]. Emerging studies suggest ethanol‐induced proinflammatory signalling alters synaptic excitability [27] and contributes to increased alcohol drinking and preference [28, 29], consistent with proinflammatory signalling altering synapses that could change alcohol responsivity and contribute to alcohol tolerance. While accumulating evidence suggests a role for alcohol‐induced neuroinflammation in alterations of alcohol drinking and AUD risk, neuroimmune system involvement in alcohol tolerance is unknown. To investigate the contributions of adolescent binge ethanol and potential neuroimmune mechanisms of AIE‐induced developmental acquisition of alcohol tolerance in adulthood, we used our ERB to assess adult acute ethanol responsivity across cumulative ethanol doses following AIE (Experiment 1), an adolescent neuroinflammatory lipopolysaccharide (LPS) challenge (Experiment 2) and post‐AIE treatment with the HMGB1‐specific inhibitor glycyrrhizic acid (Experiment 3). Given that our prior ERB studies did not find major sex differences, we focus exclusively on females in the current investigation to reduce biological heterogeneity and maximize power for the central mechanistic question—whether AIE confers persistent adult ethanol tolerance via HMGB1‐mediated neuroimmune signalling. The results from these studies support the hypothesis that proinflammatory HMGB1 signalling induces adult ethanol tolerance that is reversible, consistent with proinflammatory activation as a key target to reduce risk for AUD [30].

## 2 Methods and Materials

### 2.1 Animals

Female Wistar rats bred at the University of North Carolina at Chapel Hill were used in this study. Subjects were housed in same‐treatment pairs in a temperature‐ (20°C) and humidity‐controlled vivarium on a 12 h/12 h light/dark cycle (light onset at 7:00 AM) and provided ad libitum access to food and water. This study was conducted in an AAALAC‐accredited facility in strict accordance with NIH regulations for the care and use of animals in research and is compliant with the US National Research Council's ‘Guide for the Care and Use of Laboratory Animals.’ All experimental procedures reported in this study were approved by the Institutional Animal Care and Use Committee at UNC.

### 2.2 Adolescent Intermittent Ethanol Paradigm

**FIGURE 1:**
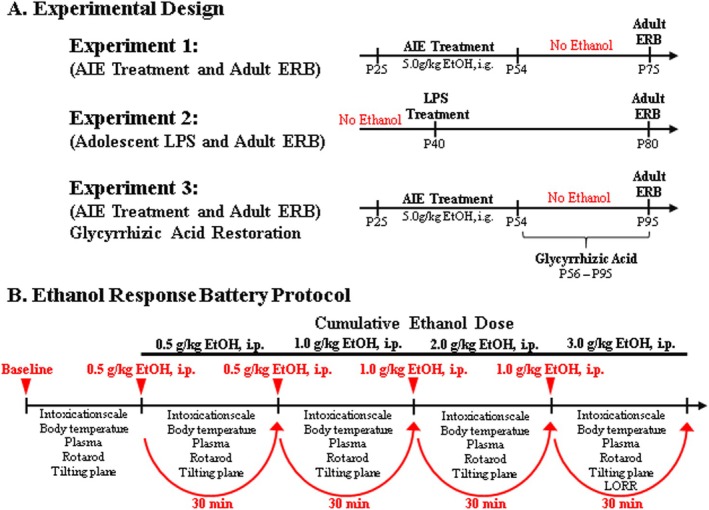
**Schematic of the experimental design and ethanol response battery (ERB) protocol.** (A) In Experiment 1, female Wistar rats received either control (CON) or adolescent intermittent ethanol (AIE) treatment from P25–P54 followed by ERB assessment on P75. In Experiment 2, ethanol‐naïve rats received a single dose of lipopolysaccharide (LPS, 1.0 mg.kg, i.p.) on P40 and were assessed on the ERB on P80. In Experiment 3, rats received CON or AIE treatment from P25–P54 followed by glycyrrhizic acid (HMGB1 inhibitor) treatment from P56–P95, and assessment of ERB performance on P95. Body weights were assessed throughout experimentation. Tail blood was collected from AIE‐ and CON‐treated subjects to assess blood ethanol concentrations (BECs) using a GM7 Analyser (Analox; London, UK) 1 h after treatment on P38 (AIE/VEH: 141 ± 22 mg/dL; AIE/glyz acid: 170 ± 18 mg/dL) and P54 (AIE/VEH: 187 ± 24 mg/dL; AIE/glyz acid: 195 ± 40 mg/dL). **(B)** Each trial of the ERB consisted of (1) the 6‐point behavioural intoxication rating scale, (2) body temperature assessment, (3) tail blood collection, (4) accelerating rotarod assessment (Experiments 1 and 2 only), (5) tilting plane assessment and (6) loss of righting reflex (LORR) following the final ethanol dose. The ERB was conducted during a cumulative ethanol dose–response challenge. Following baseline ERB assessment, rats received ethanol doses (0.5, 0.5, 1.0, 1.0 g/kg, i.p.) approximately 30 min apart for cumulative ethanol doses of 0.5, 1.0, 2.0 and 3.0 g/kg with the ERB initiated 15 min following each ethanol dose.

On P21, female Wistar rats (*N* = 56) were randomly assigned to either (i) AIE (*n* = 28 [Experiment 1: *n* = 8; Experiment 3: *n* = 20]) or (ii) water control (CON; *n* = 28 [Experiment 1: *n* = 8; Experiment 3: *n* = 20]) treatments. To minimize the impact of litter variables, no more than one subject from a given litter was assigned to a single experimental condition. From P25 to P54, AIE rats received a single daily intragastric (i.g.) administration of ethanol (5.0 g/kg, 25% EtOH, w/v) on a 2‐day on/2‐day off schedule, and CONs received an equivalent volume of water on an identical schedule as previously described [31]. Subjects in Experiment 1 underwent ERB assessment on P75 and subjects in Experiment 3 were assessed on the ERB on P95 (Figure 1A).

### 2.3 Adolescent LPS Treatment

In Experiment 2, female Wistar rats (*N* = 16; *n* = 8/group) received either a single injection of LPS (1.0 mg/kg, i.p. in sterile 0.9% saline; *E. Coli* , serotype 0111:B4; Sigma‐Aldrich, Cat. #L3024) or a comparable volume of vehicle on P40. Subjects underwent ERB assessment on P80 (Figure 1A).

### 2.4 Glycyrrhizic Acid Treatment

Adult CON‐ and AIE‐treated rats in Experiment 3 received post‐AIE treatment with glycyrrhizic acid (1.0 g/L, P.O.; Sigma‐Aldrich; Cat. #50531) or vehicle (water) in cage water bottles from P56 to P95. Subjects in the glycyrrhizic acid condition consumed an average of 37.4 mL/day with an approximate average consumption of 140 mg/kg glycyrrhizic acid per day.

### 2.5 Ethanol Response Battery

The ERB was performed as described previously [14]. The four consecutive doses of ethanol (i.e., 0.5, 0.5, 1.0, 1.0 g/kg, i.p.) produce cumulative ethanol doses of approximately 0.5, 1.0, 2.0 and 3.0 g/kg (see Figure 1B). Each subsequent ERB assessment was conducted approximately 30 min apart, each initiated 15 min after ethanol dosing. The ERB consisted of (1) the behavioural intoxication rating scale, (2) body temperature assessment, (3) tail blood collection, (4) rotarod balance (Experiments 1 and 2 only), (5) tilting plane motor coordination and (6) LORR following the final ethanol dose.

#### 2.5.1 Behavioural Intoxication Rating Scale

The 6‐point behavioural intoxication rating scale was conducted as previously described [14]. Briefly, animals are scored by two researchers according to the following behavioural scale: (1) no sign of intoxication; (2) hypoactivity; (3) slight intoxication (ataxia; slight motor impairment); (4) moderate intoxication (obvious motor impairment; dragging abdomen); (5) high intoxication (dragging abdomen; LORR); (6) extreme intoxication (LORR; loss of eye blink response)

#### 2.5.2 Body Temperature

Body temperature was assessed using a Thermalert clinical monitoring thermometer (Physitemp, Clifton, NJ, USA) with an electric thermometer probe inserted approximately 5 mm into the rectum and left in place for ≥ 45 s until a stable reading was obtained. Animals were briefly restrained for 2 min in a DecapiCone (ThermoFisher Scientific, Austin, TX, USA), and body temperature was assessed at baseline and again following each ethanol dose following completion of the behavioural intoxication rating scale for a total of five assessments. Difference (Δ) in body temperature as a consequence of cumulative ethanol dosing was calculated by subtracting body temperature following each ethanol dose from baseline body temperature. Room temperature was monitored daily and averaged 20.5°C (range 20°C–21°C).

#### 2.5.3 Tail Blood Collection

Tail blood was collected at baseline and 15 min after each ethanol administration following body temperature assessment to determine BECs using a GM7 Analyzer (Analox; London, UK) and for ELISA analysis of plasma levels of HMGB1.

#### 2.5.4 Accelerating Rotarod

The accelerating rotarod provides a measure of balance across cumulative doses of ethanol. Rats were trained on the accelerating rotarod (IITC Life Sciences, Woodland Hills, CA, USA) for 2 days (3 trials per day) prior to ERB assessment. On training day 1, each animal received three 3‐min training trials with Trial 1 at 5 rotations per minute (rpm), Trial 2 at 10 rpm and Trial 3 consisting of a start speed of 5 rpm and accelerating to 20 rpm over 3 min. Twenty‐four hours later on training day 2, each animal then received three 3‐min training trials with Trial 1 at 10 rpm followed by two consecutive trials with the rotarod cylinder with a start speed of 5 rpm and accelerating to 20 rpm over 3 min.

Twenty‐four hours later, rotarod performance was assessed during the ERB, and each session consisted of three successive trials with a start speed of 5 rpm and accelerating to 20 rpm over 3 min. Latency to fall was recorded for every trial. Each of the three successive trials per dosing session was averaged and change from baseline (Δ) in time spent on the rotarod as a consequence of cumulative ethanol dosing was calculated by subtracting time on the rotarod during baseline performance from each ethanol dose rotarod performance.

#### 2.5.5 Tilting Plane

The tilting plane assesses motor coordination, which provides a measure of the ability of rodents to maintain balance as the angle of the horizontal plane is gradually increased. The tilting plane apparatus consisted of a clear Plexiglas box (60 cm × 24 cm × 20 cm) attached with a hinge to a frame with a glass panel floor. The rat was placed on the panel and the panel lifted slowly until the subject began to slide down the floor of the apparatus. The angle at which the rat began to slide was measured across three consecutive trials per session. The three trials were averaged and the difference (Δ) in angle of slide as a consequence of cumulative ethanol dosing was calculated by subtracting angle of slide from the averaged baseline angle of slide from each ethanol dose angle of slide.

#### 2.5.6 Loss of Righting Reflex

Loss of righting reflex was assessed following the final dose of ethanol after completion of the tilting plane. Animals were placed on their back in a V‐shaped trough and assessed for righting reflex. Loss of righting reflex was defined as the inability of the rat to right itself onto all four paws within 60 s.

### 2.6 HMGB1 ELISA

Blood samples collected into heparin‐containing tubes during the tail blood collection phase of the ERB were centrifuged (2000 × g for 20 min) and plasma assessed for HMGB1. HMGB1 ELISA was performed according to the manufacturer's protocol (IBL International, Hamburg, Germany, Cat. #ST51011).

### 2.7 Immunohistochemistry, Microscopic Quantification and Image Analysis

Following the conclusion of ERB assessment in Experiment 3, animals were anaesthetised with sodium pentobarbital (100 mg/kg, i.p.), transcardially perfused with 0.1 M PBS followed by 4.0% paraformaldehyde. Brains were excised and postfixed in 4.0% paraformaldehyde for 24 h at 4°C followed by a 4‐day fixation in 30% sucrose solution. Coronal sections were cut (40 μm) on a sliding microtome (MICROM HM450; ThermoScientific, Austin, TX, USA), and sections sequentially collected into well plates and stored at −20°C in a cryoprotectant solution (30% glycol/30% ethylene glycol in PBS).

Immunohistochemistry was performed as previously described [31]. Briefly, free‐floating tissue containing the motor cortex (every 6th section; approximate Bregma: 1.70 to −0.12 mm based on the atlas of Paxinos and Watson [32]) was incubated for 48 h at 4°C in a primary antibody solution containing blocking solution with either rabbit anti‐HMGB1 (1:1000; Abcam, Cambridge, MA, USA, Cat. #ab18256), rabbit anti‐RAGE (1:1000; Abcam, Cat. #ab3611) or rabbit anti‐pNF‐κB p65 (1:2000; Abcam, Cat. #ab86299). Sections were washed in PBS, incubated for 1 h in a biotinylated secondary antibody (Vector Laboratories) and incubated for 1 h in an avidin‐biotin complex solution (1:200; Vector Laboratories). The chromogen nickel‐enhanced diaminobenzidine (Sigma‐Aldrich) was used to visualize immunoreactivity. Tissue was mounted onto slides, dehydrated and cover‐slipped.

BioQuant Nova Advanced Image Analysis software (R&M Biometric, Nashville, TN, USA) was used for image capture and quantification of immunohistochemistry. A modified unbiased stereological quantification approach was used, which was performed by experimenters blind to treatment, to quantify HMGB1 + IR, RAGE + IR and pNF‐κB p65 + IR cells in the motor cortex. We previously reported that comparison of traditional unbiased stereological methodology with our modified unbiased stereological approach yielded nearly identical values for heterogeneously distributed cell populations [33]. The outlined regions of interest were determined and data expressed as cells/mm^2^.

### 2.8 Statistical Analysis

Statistical analysis was performed using SPSS (IBM; Chicago, IL, USA) and GraphPad Prism 8 (San Diego, CA, USA). BECs, intoxication rating, body temperature, rotarod, tilting plane and HMGB1 plasma data were first assessed using the Shapiro–Wilk test to determine normality of data distribution. Data with a normal distribution were assessed using parametric two‐way mixed ANOVAs (condition × dose [repeated measure]) or three‐way mixed ANOVAs (condition × drug × dose [repeated measure]) with post hoc Bonferroni corrections or Šidák's MCT and partial *ƞ* ^2^ for omnibus terms when appropriate as described in the Results. Data that were not normally distributed was assessed using the nonparametric Mann–Whitney test. Ordinal data (i.e., intoxication rating) was assessed using the Mann–Whitney test (Experiments 1 and 2) or the Kruskal–Wallis *H* test (Experiment 3). Baseline threshold assessments were analysed using one‐way ANOVAs with post hoc Bonferroni corrections. Since our a priori hypothesis specified that glycyrrhizic acid would reverse AIE‐induced reductions in alcohol responsivity and increases in plasma HMGB1 (Experiment 3), we defined two orthogonal planned contracts: (i) CON versus AIE under the vehicle condition, and (ii) AIE/VEH versus AIE/glyz acid. These planned contrasts were evaluated irrespective of the interaction term, with Holm–Šidák adjustment across the two contrasts. We report estimated marginal mean differences (EMM Δ) with 95% confidence intervals (CIs) and partial *ƞ* ^2^ for omnibus terms. Pearson chi‐square was used to analyse LORR data on the final dose of the ERB. Immunohistochemistry data (Experiment 3) was assessed using a 2 × 2 ANOVA (condition × drug) with post hoc Bonferroni corrections and partial *ƞ* ^2^ for omnibus terms when appropriate as described in the Results. All values except intoxication rating and LORR are reported as mean ± SEM The intoxication rating data are reported as median with the interquartile range whereas LORR is reported as number of subjects with LORR.

## 3 Results

### 3.1 Adolescent Binge Ethanol Exposure Confers Long‐Lasting Low Ethanol Responsivity Across a Cumulative Ethanol Challenge in Adulthood

**FIGURE 2:**
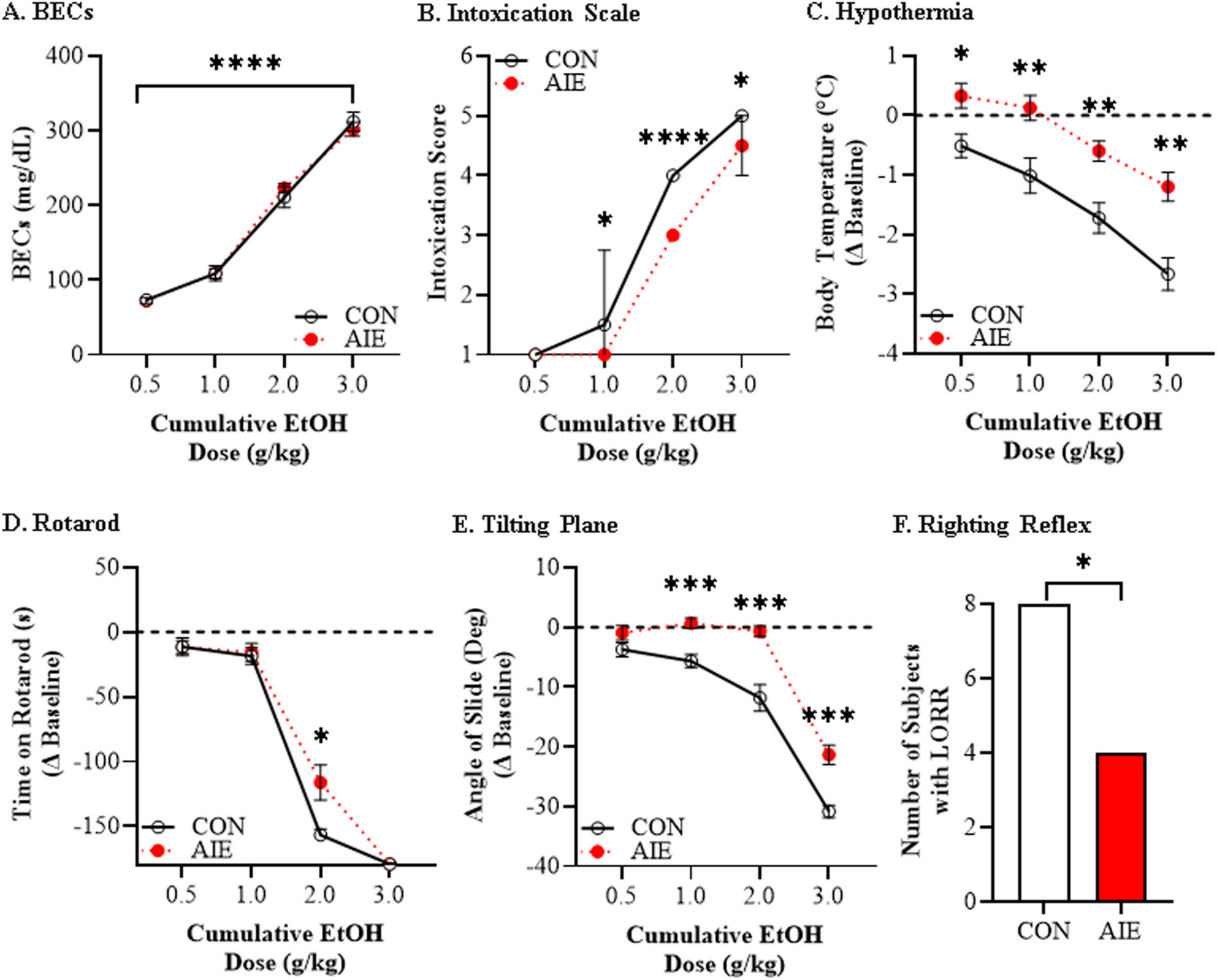
**Adolescent intermittent ethanol (AIE) confers long‐lasting low ethanol responsivity on the ethanol response battery (ERB) in adulthood.** (A) Assessment of BECs 15 min after each ethanol dose across the cumulative ethanol challenge during the ERB that did not differ as a function of AIE treatment. Data are presented as mean ±SEM. (B) Assessment of intoxication rating across the cumulative ethanol challenge revealed increasing intoxication scores across treatment conditions. Prior AIE treatment significantly decreased adult intoxication scores at 1.0, 2.0 and 3.0 g/kg relative to age‐matched CONs. Data are presented as median with the interquartile range. (C) Across adult CON‐ and AIE‐treated female rats, assessment of body temperature during the cumulative ethanol challenge revealed increasing hypothermia with rising BECs. Furthermore, AIE blunted the hypothermic response at 0.5, 1.0, 2.0 and 3.0 g/kg relative to age‐matched CONs. Data are presented as mean ±SEM. (D) Assessment of rotarod balance revealed a dose‐dependent impairment in time to remain on the rotarod across cumulative ethanol doses. Relative to age‐matched adult CONs, adult AIE‐treated rats spent significantly more time on the rotarod at the 2.0 g/kg dose. Data are presented as mean ±SEM. (E) Assessment of tilting plane motor coordination in adult female rats revealed a dose‐dependent reduction in the angle of slide across adult CON‐ and AIE‐treated rats across cumulative ethanol doses. Prior AIE treatment increased the angle of slide at 1.0, 2.0 and 3.0 g/kg) relative to age‐matched CONs. Data are presented as mean ±SEM. (F) Assessment of loss of righting reflex (LORR) following the final dose of ethanol (i.e., 3.0 g/kg) revealed all adult CON subjects displays LORR whereas four of the eight adult AIE‐treated rats demonstrated a LORR. Data are presented as number of subjects with LORR. *n* = 8 subjects/condition. *p < 0.05, **p < 0.01, ***p < 0.001, ****p < 0.0001.

Assessment of BECs during cumulative ethanol doses revealed similar progressive increases, ranging from approximately 72 (0.5 g/kg) to 307 mg/dL (3.0 g/kg) (main effect of dose: *F*[3, 42] = 473.5, *p* < 0.0001, *ƞ* ^2^ = 0.97, 2‐way mixed ANOVA) that did not differ as a function of AIE treatment (Figure 2A and Figure S1A in Supplement 1). Both CON‐ and AIE‐treated adult (i.e., P75) rats evidenced increasing intoxication scores with rising BECs (Figure 2A,B). Prior AIE treatment significantly decreased intoxication scores at 1.0 g/kg (*U* = 16.0, *p* = 0.027, Mann–Whitney), 2.0 g/kg (*U* = 0.00, *p* = 0.0002, Mann–Whitney) and 3.0 g/kg (*U* = 16.0, *p* = 0.027, Mann–Whitney) in adulthood relative to age‐matched CONs (Figure 2B). Both CON‐ and AIE‐treated adult rats evidenced increasing hypothermia (main effect of dose: *F*[3, 42] = 105.2, *p* < 0.0001, *ƞ* ^2^ = 0.88, 2‐way mixed ANOVA; Figure 2C and Figure S1B in Supplement 1) across cumulative ethanol doses. Post hoc analysis of the significant dose × treatment interaction (*F*[3, 42] = 3.8, *p* = 0.024, *ƞ* ^2^ = 0.21, 2‐way mixed ANOVA) revealed that AIE blunted the hypothermic response at 0.5 g/kg (*F*[1, 14] = 8.5, *p* = 0.011, *ƞ* ^2^ = 0.38, Bonferroni), 1.0 g/kg (*F*[1, 14] = 9.9, *p* = 0.007, *ƞ* ^2^ = 0.41, Bonferroni), 2.0 g/kg (*F*[1, 14] = 13.8, *p* = 0.002, *ƞ* ^2^ = 0.50, Bonferroni) and 3.0 g/kg (*F*[1, 14] = 16.0, *p* = 0.001, *ƞ* ^2^ = 0.53, Bonferroni) relative to age‐matched CONs (Figure 2C). Furthermore, adult CONs evidenced a significant baseline hypothermic threshold shift at the 1.0 g/kg dose (*p* = 0.0009, *ƞ* ^2^ = 0.82, 1‐way ANOVA with Bonferroni), whereas AIE‐treated animals evidenced a significant baseline threshold difference at the 3.0 g/kg dose (*p* = 0.0004, *ƞ* ^2^ = 0.58, 1‐way ANOVA with Bonferroni). Time on the accelerating rotarod progressively decreased as ethanol dose increased (main effect of dose: *F*[3, 42] = 381.1, *p* < 0.0001, *ƞ* ^2^ = 0.97, 2‐way mixed ANOVA; Figures 2D and S1C in Supplement 1). Post hoc assessment of the significant dose × treatment interaction (*F*[3, 42] = 5.2, *p* = 0.004, *ƞ* ^2^ = 0.27, 2‐way mixed ANOVA) revealed that AIE significantly increased time on the rotarod in adulthood at 2.0 g/kg (*F*[1, 14] = 8.1, *p* = 0.013, *ƞ* ^2^ = 0.37, Bonferroni) relative to CONs. Adult CONs evidenced a significant baseline rotarod threshold shift at 1.0 g/kg (*p* = 0.037, *ƞ* ^2^ = 0.97, 1‐way ANOVA with Bonferroni), whereas AIE‐treated animals evidenced a significant baseline threshold shift at the 2.0 g/kg dose (*p* < 0.0001, *ƞ* ^2^ = 0.94, 1‐way ANOVA with Bonferroni). Angle of slide on the tilting plane dose‐dependently decreased across cumulative ethanol doses (main effect of dose: *F*[3, 42] = 191.2, *p* < 0.0001, *ƞ* ^2^ = 0.93, 2‐way mixed ANOVA; Figure 2E and Figure S1D in Supplement 1). Post hoc analysis of the significant dose × treatment interaction (*F*[3, 42] = 5.0, *p* = 0.004, *ƞ* ^2^ = 0.27, 2‐way mixed ANOVA) revealed that AIE increased the angle of slide at 1.0 g/kg (*F*[1, 14] = 20.5, *p* = 0.0005, *ƞ* ^2^ = 0.59, Bonferroni), 2.0 g/kg (*F*[1, 14] = 21.5, *p* = 0.0004, *ƞ* ^2^ = 0.61, Bonferroni) and 3.0 g/kg (*F*[1, 14] = 24.7, *p* = 0.0002, *ƞ* ^2^ = 0.64, Bonferroni) relative to CONs. Furthermore, adult CONs evidenced a significant baseline tilting plane threshold shift at the 1.0 g/kg dose (*p* = 0.043, *ƞ* ^2^ = 0.89, 1‐way ANOVA with Bonferroni), whereas AIE‐treated animals evidenced a significant baseline threshold shift at the 3.0 g/kg dose (*p* < 0.0001, *ƞ* ^2^ = 0.90, 1‐way ANOVA with Bonferroni). Assessment of LORR revealed that all adult CON subjects (*n* = 8) displayed LORR whereas only 4 of the 8 adult AIE‐treated rats demonstrated a LORR (χ^2^(1, *N* = 16) = 5.3, *p* = 0.021, Pearson chi‐square; Figure 2F). Thus, adult AIE‐treated rats evidenced lower intoxication scores, decreased hypothermia and diminished impairment of balance and motor coordination across a broad range of acute cumulative ethanol doses.

**FIGURE 3:**
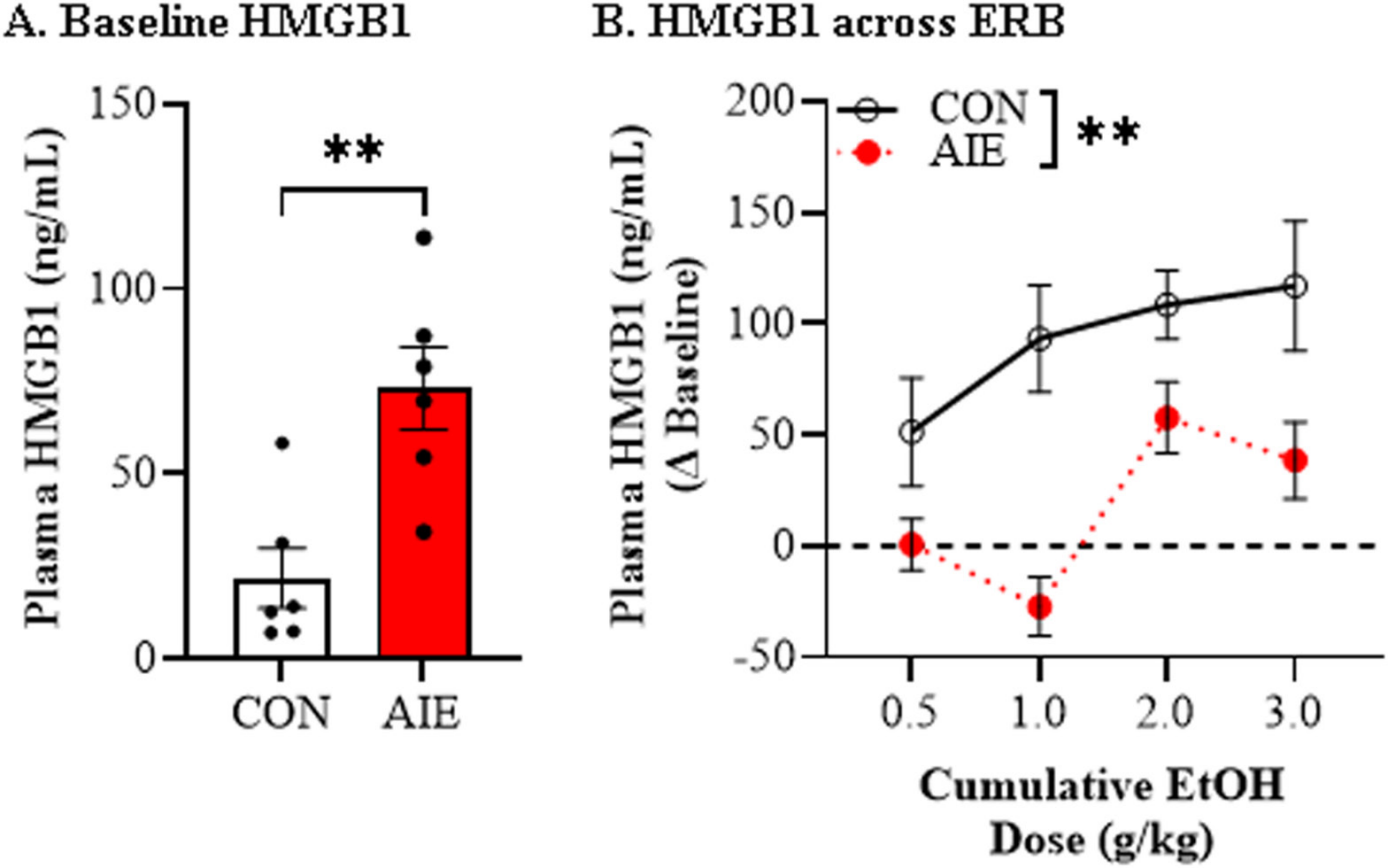
**Adolescent intermittent ethanol (AIE) alters adult plasma high mobility group box 1 (HMGB1) responsivity to a cumulative ethanol challenge during ethanol response battery (ERB) assessment in adulthood.** (A) Assessment of baseline plasma HMGB1 levels prior to ERB assessment in adulthood revealed a persistent AIE‐induced 3.4‐fold increase relative to age‐matched CONs. (B) Across cumulative ethanol doses of the ERB, plasma levels of HMGB1 increased in a dose‐dependent manner in CON‐ and AIE‐treated adult rats. In adult rats with a history of AIE exposure, ERB assessment revealed a significant reduction of plasma HMGB1 across ERB testing relative to age‐matched CONs. *n* = 6 subjects/condition. Data are presented as mean ±SEM. *p < 0.05, **p < 0.01.

High mobility group box 1 (HMGB1) is a proinflammatory cytokine‐like protein released by ethanol in neuronal, microglial and astrocyte cultures as well as in forebrain and hippocampal slice culture models [34]. We next assessed plasma levels of HMGB1 in adult animals following AIE during the cumulative ethanol challenge. Baseline HMGB1 plasma levels were increased in AIE‐treated animals relative to CONs (*t* [10] = 3.7, *p* = 0.004, *ƞ* ^2^ = 0.58, Student's *t* test; Figure 3A). Across the ERB, we observed a dose‐dependent increase in plasma HMGB1 (main effect of dose: *F*[3, 30] = 8.7, *p* = 0.0003, *ƞ* ^2^ = 0.47, 2‐way mixed ANOVA; Figure 3B) as well as an overall reduction of plasma HMGB1 levels in adult AIE‐treated animals across the ERB (main effect of treatment: *F*[1, 10] = 11.8, *p* = 0.006, *ƞ* ^2^ = 0.54, 2‐way mixed ANOVA). Interestingly, adult CONs evidenced a significant baseline plasma HMGB1 threshold shift at the 2.0 g/kg dose (*p* = 0.016, *ƞ* ^2^ = 0.45, 1‐way ANOVA with Bonferroni), whereas baseline threshold shifts were not observed in the adult AIE‐treated animals. These findings are consistent with AIE altering acute ethanol‐induced release of HMGB1.

### 3.2 An Adolescent Neuroimmune Challenge With LPS Confers Long‐Lasting Low Ethanol Responsivity to a Cumulative Ethanol Challenge in Adulthood

**FIGURE 4:**
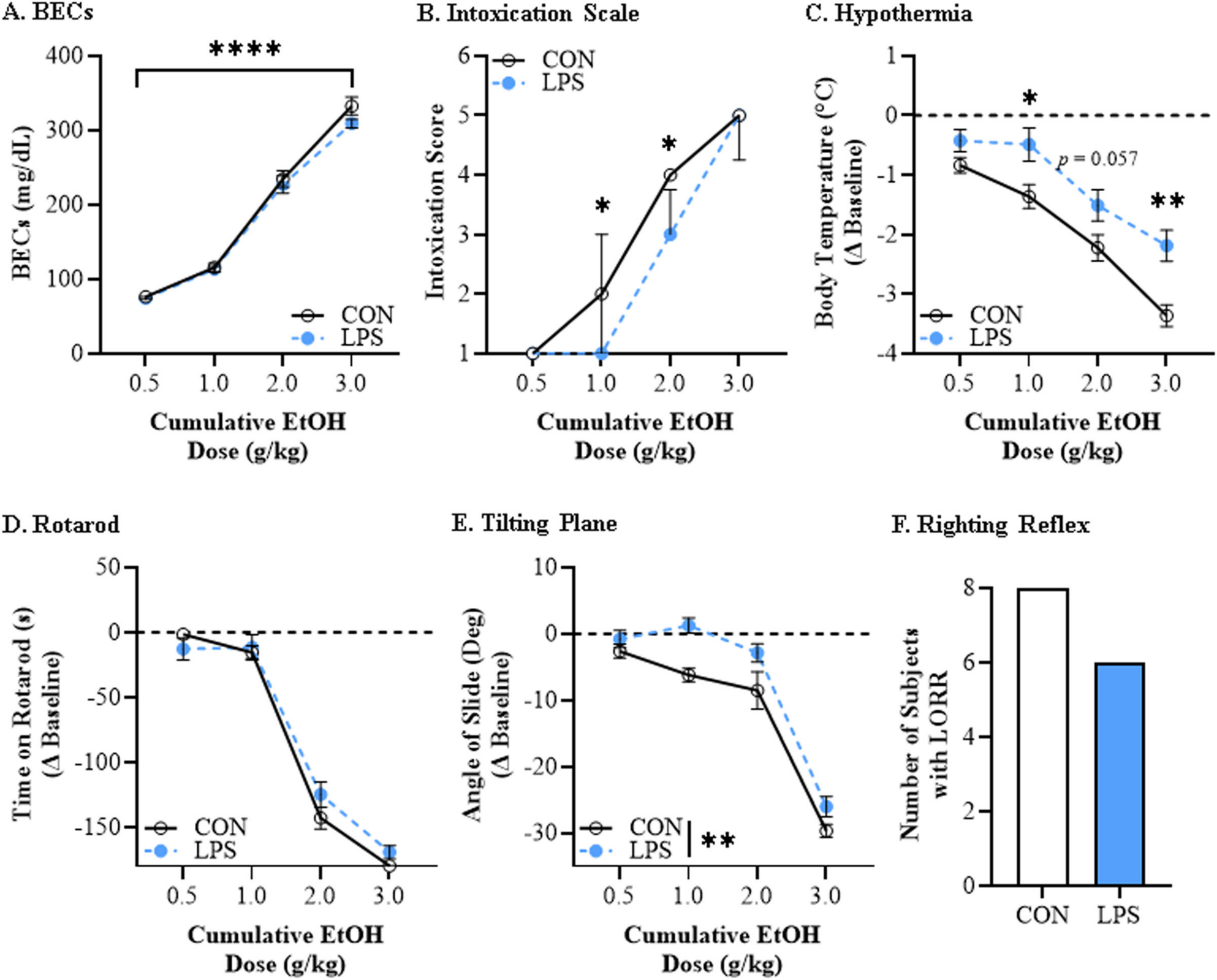
**Adolescent lipopolysaccharide (LPS) exposure confers long‐lasting low ethanol responsivity on the ethanol response battery (ERB) in adulthood.** (A) Assessment of BECs 15 min after each ethanol dose across the cumulative ethanol challenge during the ERB revealed a dose‐dependent increase in BECs that did not differ as a function of prior LPS exposure. Data are presented as mean ±SEM. (B) Intoxication rating assessment across the cumulative ethanol challenge revealed increasing intoxication scores in adult CON‐ and LPS‐treated rats. Adolescent LPS treatment decreased adult intoxication scores at doses of 1.0 and 2.0 g/kg relative to age‐matched CONs. Data are presented as median with the interquartile range. (C) Adult CON‐ and LPS‐treated rats evidenced an increasing hypothermic response across cumulative ethanol doses. Prior adolescent LPS treatment decreased the hypothermic response at 1.0 and 3.0 g/kg relative to age‐matched CONs. Data are presented as mean ±SEM. (D) Assessment of balance on the rotarod revealed that latency to fall decreased, regardless of treatment condition, across cumulative ethanol doses while prior LPS exposure did not affect rotarod performance. Data are presented as mean ±SEM. (E) Assessment of tilting plane motor coordination in adult female rats revealed a dose‐dependent reduction in the angle of slide across adult CON‐ and LPS‐treated rats. Adolescent LPS treatment led to an overall decrease in tilting plane impairment in adulthood relative to age‐matched CONs. Data are presented as mean ±SEM. (F) Assessment of loss of righting reflex (LORR) following the final dose of ethanol (i.e., 3.0 g/kg) revealed all adult CON subjects displays LORR whereas six of the eight adult LPS‐treated rats demonstrated a LORR. Data are presented as number of subjects with LORR. *n* = 8 subjects/condition. *p < 0.05, **p < 0.01, ****p < 0.0001.

To determine if adolescent proinflammatory gene induction alters adult acute alcohol responsivity, we treated adolescent rats with LPS on P40 and assessed ERB performance 40 days later on P80. Across the ERB, BECs progressively increased (main effect of dose: *F*[3, 42] = 529.3, *p* < 0.0001, *ƞ* ^2^ = 0.97, 2‐way mixed ANOVA) that did not differ as a function of adolescent LPS treatment (Figure 4A). Adolescent LPS significantly blunted adult intoxication scores at doses of 1.0 g/kg (*U* = 14.5, *p* = 0.033, Mann–Whitney) and 2.0 g/kg (*U* = 12, *p* = 0.015, Mann–Whitney) relative to CONs (Figure 4B). Assessment of body temperature during adult ERB testing revealed an increasing hypothermic response across cumulative ethanol doses (main effect of dose: *F*[3, 42] = 143.0, *p* < 0.0001, *ƞ* ^2^ = 0.91, 2‐way mixed ANOVA; Figure 4C and Figure S2B in Supplement 1). Post hoc analysis of the significant dose × treatment interaction (*F*[3, 42] = 3.8, *p* = 0.016, *ƞ* ^2^ = 0.22, 2‐way mixed ANOVA) revealed that adolescent LPS decreased the adult hypothermic response at 1.0 g/kg (*F*[1, 14] = 6.5, *p* = 0.024, *ƞ* ^2^ = 0.32, Bonferroni) and 3.0 g/kg (*F*[1, 14] = 13.8, *p* = 0.002, *ƞ* ^2^ = 0.50, Bonferroni) relative to CONs. Furthermore, adult CONs evidenced a significant baseline hypothermic threshold shift at the 0.5 g/kg dose (*p* = 0.009, *ƞ* ^2^ = 0.88, 1‐way ANOVA with Bonferroni), whereas LPS‐treated animals evidenced a significant baseline threshold shift at the 2.0 g/kg dose (*p* < 0.0001, *ƞ* ^2^ = 0.76, 1‐way ANOVA with Bonferroni). Interestingly, while adult CON‐ and LPS‐treated animals evidenced dose‐dependent decreases in time on the rotarod (main effect of dose: *F*[3, 42] = 386.6, *p* < 0.0001, *ƞ* ^2^ = 0.97, 2‐way mixed ANOVA; Figure 4D and Figure S4C in Supplement 1), we did not observe alterations in rotarod balance due to adolescent LPS exposure. Assessment of motor coordination on the tilting plane revealed a dose‐dependent reduction in angle of slide across cumulative ethanol doses in CON‐ and LPS‐treated rats (main effect of dose: *F*[3, 42] = 189.1, *p* < 0.0001, *ƞ* ^2^ = 0.93, 2‐way mixed ANOVA; Figure 4E and Figure S4D in Supplement 1). Interestingly, adolescent LPS decreased overall tilting plane impairment in adulthood relative to CONs (main effect of treatment: *F*[1, 14] = 10.0, *p* = 0.007, *ƞ* ^2^ = 0.42, 2‐way mixed ANOVA). Furthermore, adult CONs evidenced a significant baseline tilting plane threshold shift at the 2.0 g/kg dose (*p* = 0.002, *ƞ* ^2^ = 0.89, 1‐way ANOVA with Bonferroni), whereas LPS‐treated animals evidenced a significant baseline threshold shift at the 3.0 g/kg dose (*p* < 0.0001, *ƞ* ^2^ = 0.92, 1‐way ANOVA with Bonferroni). Assessment of LORR revealed that all adult CON subjects displayed LORR whereas six of the eight adult LPS‐treated rats demonstrated a LORR (χ^*2*^(1, *N* = 16) = 2.3, *p* = 0.131, Pearson chi‐square; Figure 4F).

**FIGURE 5:**
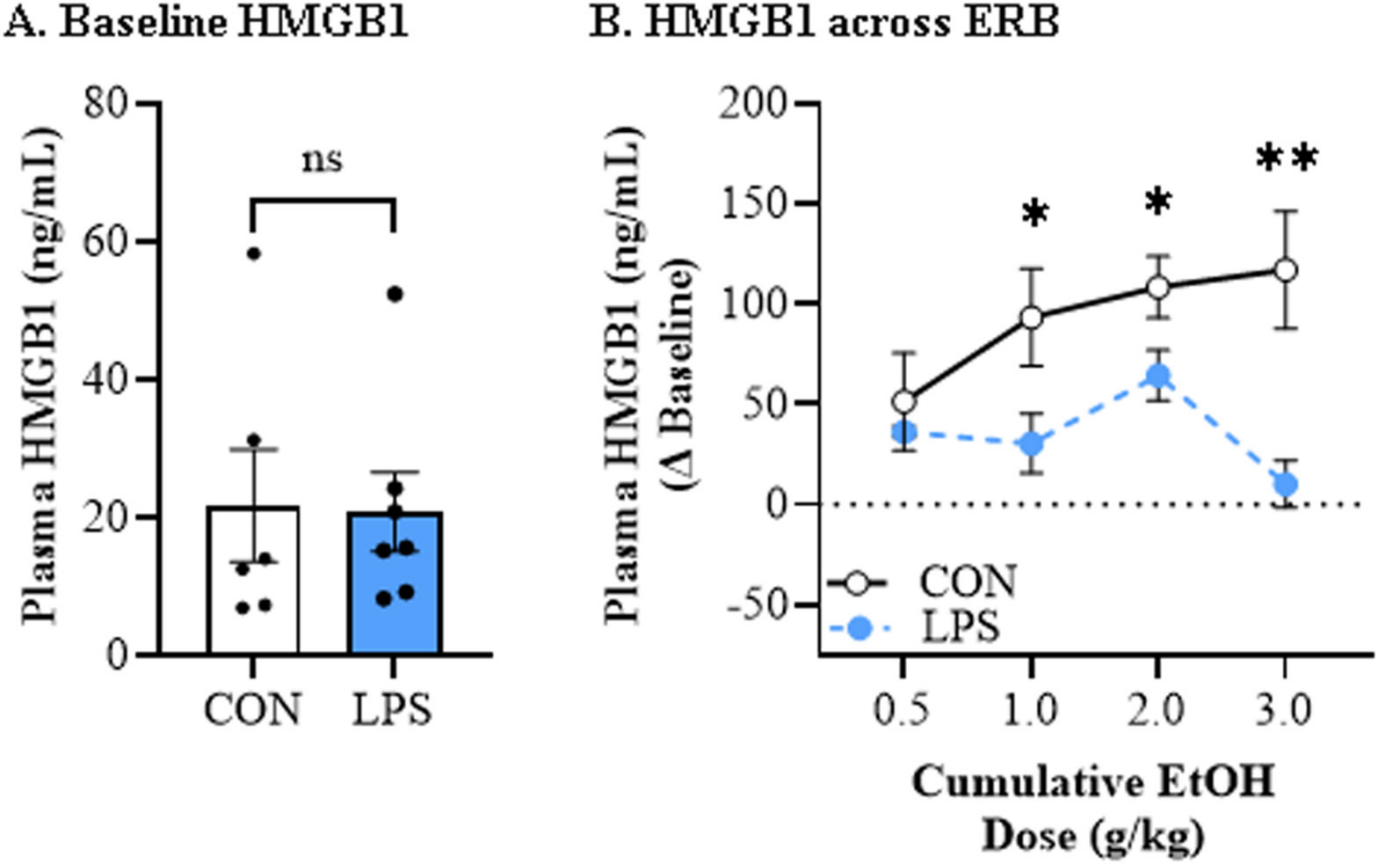
**Adolescent lipopolysaccharide (LPS) exposure alters adult plasma high mobility group box 1 (HMGB1) responsivity to a cumulative ethanol challenge during ethanol response battery (ERB) assessment in adulthood.** (A) Baseline HMGB1 plasma levels did not differ between treatment groups prior to ERB assessment in adulthood. (B) Across cumulative ethanol doses of the ERB, plasma levels of HMGB1 increased in a dose‐dependent manner in CON‐ and adolescent LPS‐treated adult rats. In adult rats with a history of adolescent LPS exposure, ERB assessment revealed a reduction in plasma HMGB1 levels at ethanol doses of 1.0, 2.0 and 3.0 g/kg relative to age‐matched CONs. *n* = 6 subjects/condition. Data are presented as mean ±SEM. *p < 0.05, **p < 0.01.

Plasma HMGB1 levels at baseline did not differ between CON‐ and LPS‐treated adult rats (Figure 5A). Across cumulative ethanol doses, we observed a dose‐dependent increase in plasma HMGB1 levels (main effect of dose: *F*[3, 33] = 3.9, *p* = 0.017, *ƞ* ^2^ = 0.26, 2‐way mixed ANOVA; Figure 5B). Post hoc analysis of the significant dose × treatment interaction (*F*[3, 33] = 4.8, *p* = 0.007, *ƞ* ^2^ = 0.30, 2‐way mixed ANOVA) revealed an adolescent LPS‐induced reduction in plasma HMGB1 levels at ethanol doses of 1.0 g/kg (*F*[1, 11] = 5.3, *p* = 0.042, *ƞ* ^2^ = 0.32, Bonferroni), 2.0 g/kg (*F*[1, 11] = 5.0, *p* = 0.046, *ƞ* ^2^ = 0.31, Bonferroni) and 3.0 g/kg (*F*[1, 11] = 13.0, *p* = 0.004, *ƞ* ^2^ = 0.54, Bonferroni) relative to CONs. Together, these findings suggest that an adolescent neuroimmune challenge, perhaps through a HMGB1‐mediated neuroinflammatory mechanism, confers lasting alcohol tolerance similar to AIE treatment.

### 3.3 The HMGB1 Inhibitor Glycyrrhizic Acid Reverses AIE‐Induced Long‐Lasting Low Ethanol Responsivity to a Cumulative Ethanol Challenge in Adulthood

**FIGURE 6:**
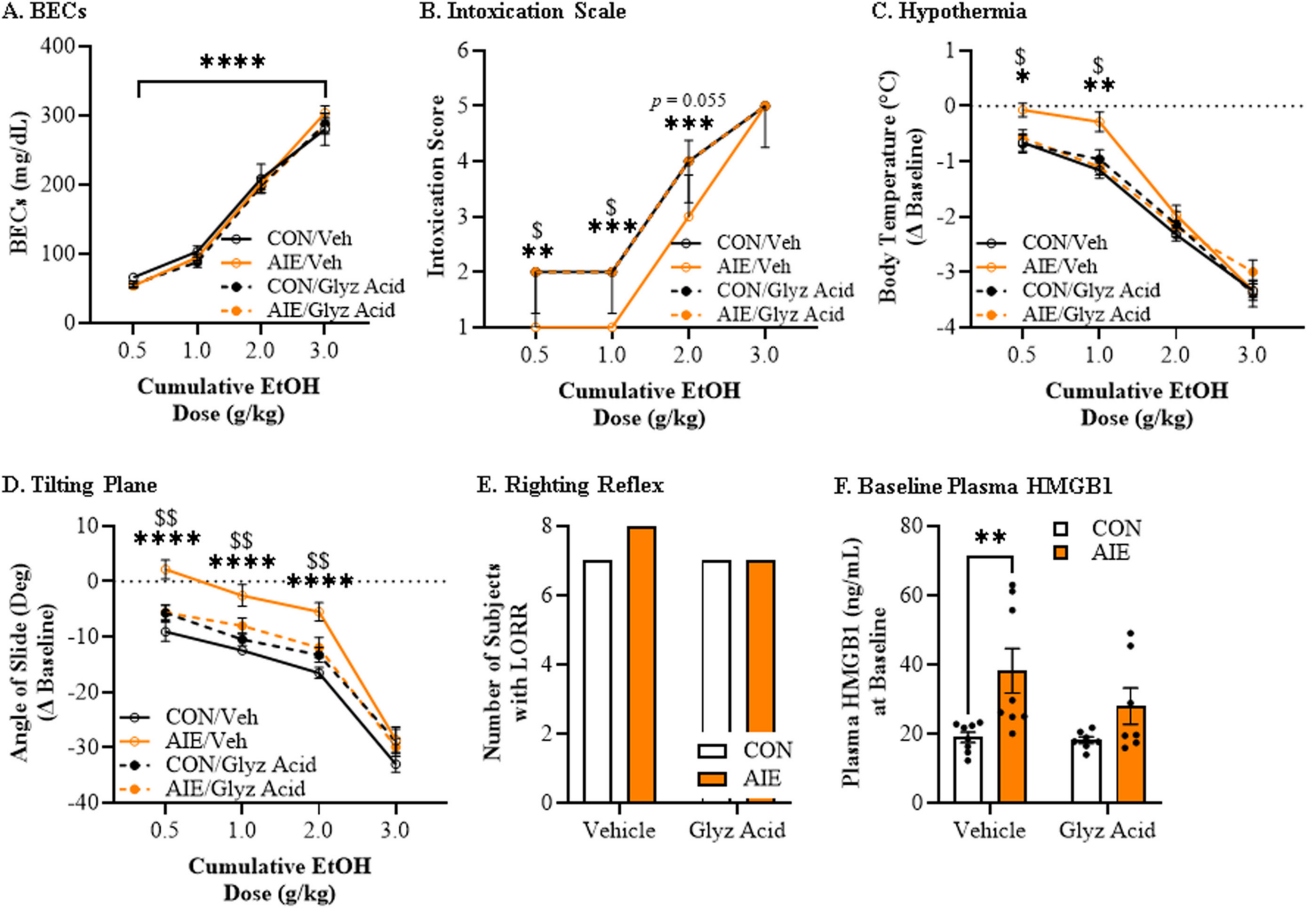
**Adult treatment with the HMGB1 inhibitor glycyrrhizic acid (glyz acid) reverses adolescent intermittent ethanol (AIE)‐induced acquisition of long‐lasting low ethanol responsivity to a cumulative ethanol challenge in adulthood.** (A) Assessment of BECs 15 min after each ethanol dose across the cumulative ethanol challenge during the ERB revealed a dose‐dependent increase in BECs that did not differ as a function of AIE or glycyrrhizic acid treatment. Data are presented as mean ±SEM. (B) Assessment of intoxication rating across the cumulative ethanol challenge revealed increasing intoxication scores, regardless of treatment condition. Prior AIE treatment significantly decreased adult intoxication scores at 0.5, 1.0 and 2.0 g/kg relative to age‐matched vehicle‐treated CONs. Glycyrrhizic acid restored the AIE‐induced low intoxication rating to CON levels at ethanol doses of 0.5, 1.0 and 2.0 g/kg relative to age‐matched vehicle‐treated AIE animals. Data are presented as median with the interquartile range. (C) Assessment of body temperature in adulthood across the cumulative ethanol challenge revealed increasing hypothermia with rising BECs regardless of treatment. Prior AIE treatment in vehicle‐treated rats decreased the hypothermic response across the cumulative ethanol challenge at doses of 0.5 and 1.0 g/kg relative to vehicle‐treated CONs. Glycyrrhizic acid reversed the AIE‐induced blunting of hypothermia at ethanol doses of 0.5 and 1.0 g/kg relative to vehicle‐treated AIE animals. Data are presented as mean ±SEM. (D) Assessment of motor coordination on the tilting plane revealed a dose‐dependent reduction in the angle of slide across treatment groups. Prior AIE treatment in vehicle‐treated rats increased the angle of slide at 0.5, 1.0 and 2.0 g/kg relative to age‐matched vehicle‐treated CONs. Glycyrrhizic acid restored cumulative ethanol‐induced impairments in adult AIE‐treated rats at doses of 0.5, 1.0 and 2.0 g/kg relative to vehicle‐treated AIE animals. Data are presented as mean ±SEM. (E) Assessment of loss of righting reflex (LORR) following the final dose of ethanol (i.e., 3.0 g/kg) revealed no differences across treatment conditions. (F) Assessment of plasma HMGB1 levels at baseline revealed that AIE treatment increased HMGB1 plasma levels relative to CONs. Follow‐up analyses revealed significantly increased plasma HMGB1 levels in vehicle‐treated AIE rats relative to vehicle‐treated CONs while glycyrrhizic acid treatment in AIE‐treated animals blunted the increase in HMGB1 plasma levels. Data are presented as mean ±SEM. *n* = 8 subjects/condition. *p < 0.05, **p < 0.01, ***p < 0.001, ****p < 0.0001, CON/VEH versus AIE/VEH; $*p* < 0.05, $$*p* < 0.01, AIE/VEH versus AIE/glyz acid.

Glycyrrhizic acid is an HMGB1 inhibitor that binds to a central pocket on HMGB1, antagonizing activation of multiple proinflammatory receptors [35]. To determine if AIE‐induced alterations in ERB tolerance involve proinflammatory HMGB1 signalling, we administered glycyrrhizic acid from P56 (48 h post‐AIE) until ERB assessment on P95 (Figure S3A in Supplement 1). Assessment of BECs revealed similar progressive increases (main effect of dose: *F*[3, 84] = 518.9, *p* < 0.0001, *ƞ* ^2^ = 0.95, 3‐way mixed ANOVA) that did not differ across treatment (Figure 6A). Similar to Experiment 1, intoxication rating differed significantly across treatment groups with adult vehicle‐treated AIE rats demonstrating lower intoxication scores at ethanol doses of 0.5 g/kg (*p* = 0.003, Kruskal–Wallis), 1.0 g/kg (*p* = 0.0002, Kruskal–Wallis) and 2.0 g/kg (*p* = 0.0003, Kruskal–Wallis) relative to CONs. Furthermore, glycyrrhizic acid normalized the AIE‐induced low intoxication rating to CON levels at ethanol doses of 0.5 g/kg (*p* = 0.012, Kruskal–Wallis), 1.0 g/kg (*p* = 0.017, Kruskal–Wallis) and 2.0 g/kg (*p* = 0.055, Kruskal–Wallis) relative to vehicle‐treated AIE animals (Figure 6B). All rats evidenced increasing hypothermia across cumulative ethanol doses (main effect of dose: *F*[3, 84] = 331.5, *p* < 0.0001, *ƞ* ^2^ = 0.92, 3‐way mixed ANOVA) with a nonsignificant dose × treatment × drug interaction (*F*[3, 84] = 2.0, *p* = 0.114, *ƞ* ^2^ = 0.07, 3‐way mixed ANOVA). Consistent with our a priori hypothesis, vehicle‐treated AIE animals evidenced a blunted hypothermic response at ethanol doses of 0.5 g/kg (EMM Δ = −0.46, 95% CI −0.84 to −0.09, *p* = 0.02, Holm–Šidák) and 1.0 g/kg (EMM Δ = −0.75, 95% CI −1.21 to −0.29, *p* = 0.003, Holm–Šidák) relative to vehicle‐treated CONs. Post‐AIE glycyrrhizic acid reversed the AIE‐induced blunting of hypothermia at ethanol doses of 0.5 g/kg (EMM Δ = −0.46, 95% CI −0.84 to −0.09, *p* = 0.02, Holm–Šidák) and 1.0 g/kg (EMM Δ = −0.75, 95% CI −1.21 to −0.29, *p* = 0.003, Holm–Šidák) relative to vehicle‐treated AIE animals (Figure 6C and Figure S3B in Supplement 1). Motor coordination assessment on the tilting plane was similarly reduced across cumulative ethanol doses (main effect of dose: (*F*[3,84] = 336.9, *p* < 0.0001, *ƞ* ^2^ = 0.92, 3‐way mixed ANOVA) across treatment conditions with a nonsignificant dose × treatment × drug interaction (*F*[3, 84] = 1.0, *p* = 0.39, *ƞ* ^2^ = 0.04, 3‐way mixed ANOVA). Consistent with our a priori hypothesis, vehicle‐treated AIE animals evidenced blunted motor coordination impairment on the tilting plane at ethanol doses of 0.5 g/kg (EMM Δ = −11.3, 95% CI −15.8 to −6.7, *p* < 0.0001, Holm–Šidák), 1.0 g/kg (EMM Δ = −9.9, 95% CI −13.9 to −5.9, *p* < 0.0001, Holm–Šidák) and 2.0 g/kg (EMM Δ = −11.0, 95% CI −15.4 to −6.7, *p* < 0.0001, Holm–Šidák) relative to vehicle‐treated CONs. Post‐AIE glycyrrhizic acid reversed the AIE‐induced blunting of motor coordination impairment at ethanol doses of 0.5 g/kg (EMM Δ = 7.8, 95% CI 3.3 to 12.3, *p* = 0.001, Holm–Šidák), 1.0 g/kg (EMM Δ = 5.5, 95% CI 1.5 to 9.5, *p* = 0.009, Holm–Šidák) and 2.0 g/kg (EMM Δ = 6.5, 95% CI 2.1 to 10.8, *p* = 0.005, Holm–Šidák) relative to vehicle‐treated AIE animals (Figure 6D and Figure S3C in Supplement 1). In this cohort, assessment of LORR revealed no differences across treatment conditions (Figure 6E). Thus, similar to Experiment 1, adult vehicle‐treated AIE rats evidenced increased adult tolerance to acute ethanol intoxication that was reversed by treatment with the HMGB1 inhibitor glycyrrhizic acid.

Assessment of baseline plasma HMGB1 levels revealed that AIE increased HMGB1 plasma levels relative to CONs (main effect of treatment: *F*[1, 26] = 11.2, *p* = 0.003, *ƞ* ^2^ = 0.30, 2‐way ANOVA) with a nonsignificant treatment × drug interaction (*F*[1, 26] = 1.2, *p* = 0.29, *ƞ* ^2^ = 0.04, 2‐way ANOVA). Consistent with our a priori hypothesis, vehicle‐treated AIE animals evidenced an approximate 2.0‐fold increase of plasma HMGB1 (EMM Δ = −19.2, 95% CI −31.4 to −7.0, *p* = 0.003, Holm–Šidák) while post‐AIE glycyrrhizic acid blunted the AIE‐induced increase of plasma HMGB1 by approximately 26% (EMM Δ = 10.3, 95% CI −2.4 to 22.9, *p* = 0.11, Holm–Šidák) relative to vehicle‐treated AIE animals (Figure 6F).

**FIGURE 7:**
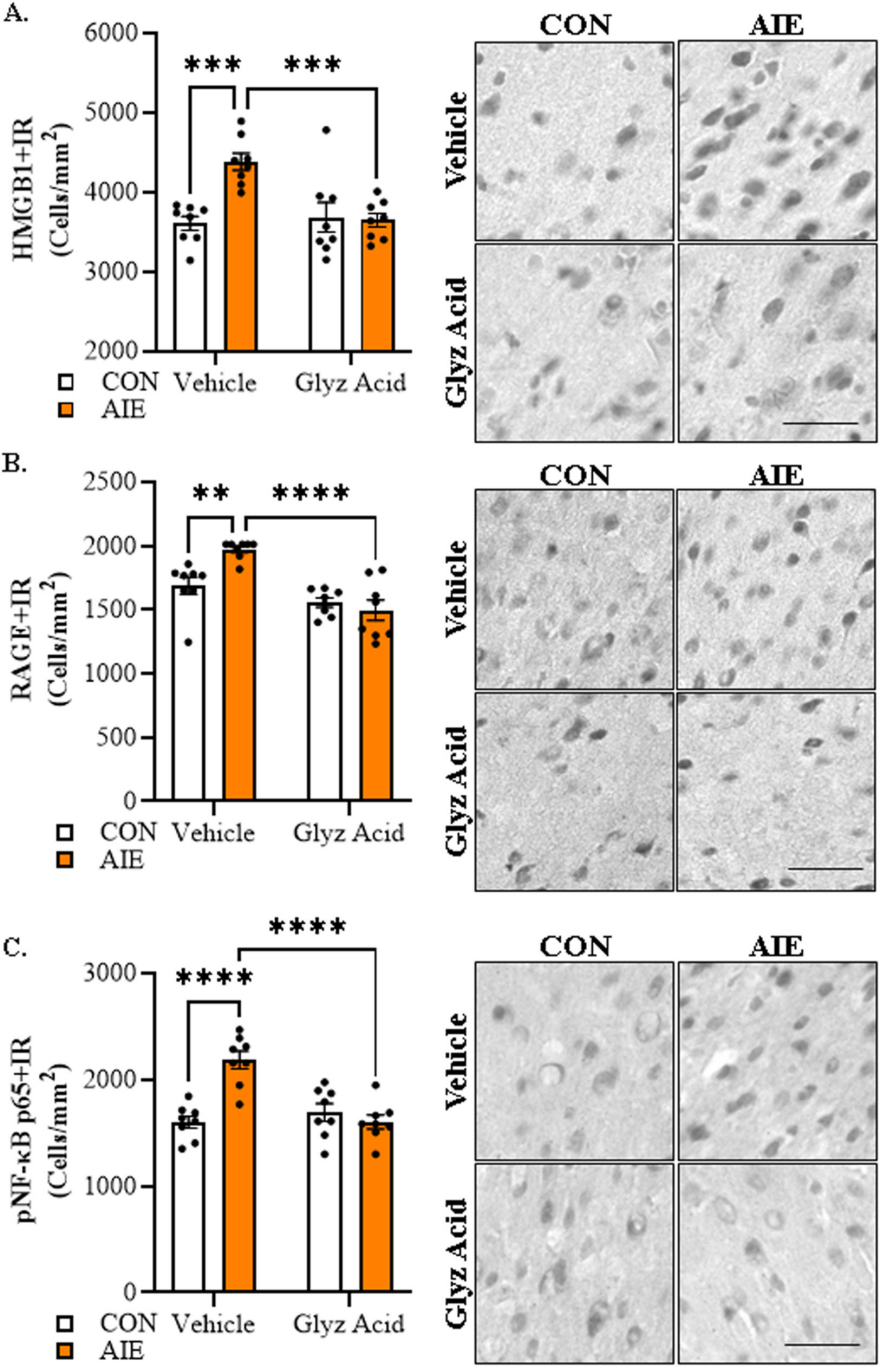
**Immunohistological assessment of proinflammatory neuroimmune markers in the adult motor cortex following AIE treatment.** (A) Immunohistochemical assessment of HMGB1 + IR in the adult motor cortex revealed an approximate 1.2‐fold increase in adult vehicle‐treated AIE animals, an effect that was reversed with glycyrrhizic acid (glyz acid). (B) Immunohistochemical assessment of RAGE + IR in the adult motor cortex revealed an approximate 1.2‐fold increase in adult vehicle‐treated AIE animals that was reversed by glycyrrhizic acid in AIE‐treated animals. (C) Immunohistochemical assessment of pNF‐κBp65 + IR in the adult motor cortex revealed an approximate 1.4‐fold increase in adult vehicle‐treated AIE animals, an effect that was reversed with glycyrrhizic acid. Scale bar = 30 μm. *n* = 8 subjects/condition. Data are presented as mean ±SEM. ***p < 0.001, ****p < 0.0001.

Previous studies have shown that AIE increases HMGB1 in the brain [26, 31]. The ERB endpoints include motor function assessments, so we performed immunohistochemistry in the M1 primary motor cortex. Immunohistochemical assessment of HMGB1 revealed a significant treatment × drug interaction (*F*[1, 28] = 10.8, *p* = 0.003, *ƞ* ^2^ = 0.28, 2‐way ANOVA). Post hoc analysis of the significant interaction revealed a 1.2‐fold increase of HMGB1 + IR in vehicle‐treated AIE animals (*F*[1, 28] = 19.7, *p* = 0.0001, *ƞ* ^2^ = 0.41, Bonferroni) that was restored to CON levels by glycyrrhizic acid (*F*[1, 28] = 17.7, *p* = 0.0002, *ƞ* ^2^ = 0.39, Bonferroni; Figure 7A). Assessment of the receptor for advanced glycation end products (RAGE), which is a receptor for HMGB1, revealed a significant treatment × drug interaction (*F*[1, 28] = 9.1, *p* = 0.005, *ƞ* ^2^ = 0.25, 2‐way ANOVA). Post hoc analysis of the significant interaction revealed a 1.2‐fold increase in vehicle‐treated AIE animals (*F*[1, 28] = 12.4, *p* = 0.001, *ƞ* ^2^ = 0.31, Bonferroni) that was reversed by glycyrrhizic acid in AIE‐treated animals (*F*[1, 28] = 34.8, *p* < 0.0001, *ƞ* ^2^ = 0.55, Bonferroni; Figure 7B). Analysis of phosphorylated (activated) NF‐κBp65, which is a transcription factor involved in activation of proinflammatory signalling, revealed a significant treatment × drug interaction (*F*[1, 28] = 21.5, *p* < 0.0001, *ƞ* ^2^ = 0.43, 2‐way ANOVA). Post hoc analysis of the significant interaction revealed a 1.4‐fold increase in vehicle‐treated AIE animals (*F*[1, 28] = 32.5, *p* < 0.0001, *ƞ* ^2^ = 0.54, Bonferroni); this increase was reversed by adult glycyrrhizic acid treatment (*F*[1, 28] = 32.2, *p* < 0.0001, *ƞ* ^2^ = 0.54, Bonferroni; Figure 7C). Together, these findings suggest that blockade of HMGB1 by glycyrrhizic acid reverses AIE‐induced neuroinflammation and persistent alcohol tolerance in adulthood.

## 4 Discussion

To our knowledge, this is the first study to link proinflammatory HMGB1 signalling to adolescent binge ethanol exposure‐induced acquisition of persistent alcohol tolerance in adulthood. The cumulative nature of the ERB allows dose–response assessments of alcohol responsivity as BECs progressively increase. We observed that BECs were remarkably similar between treatment groups and across experiments, suggesting that alterations in ethanol responsivity are not attributable to differences in ethanol metabolism. Alcohol tolerance is universally endorsed as a key symptom of AUD [2] and refers to diminished acute alcohol responsivity with continued use of the same quantity of alcohol. Although epidemiological studies link adult alcohol abuse and AUD with an adolescent age of drinking onset [4], the contribution of adolescent binge drinking to acquired tolerance in adulthood is poorly understood, due in part to the ethical considerations related to legal drinking and continued alcohol use across aging. Previous studies reported decreased alcohol responsivity in adult male rats following adolescent ethanol exposure on individual measures of motor function [15]. We replicate and extend these studies to adult female Wistar rats and report that AIE reduces cumulative ethanol dose response impairments on measures of intoxication, hypothermia, tilting plane motor coordination and LORR despite similar BECs. Furthermore, we find baseline plasma levels of HMGB1 are persistently elevated in adult AIE‐treated animals, whereas HMGB1 plasma levels are significantly blunted following AIE across cumulative ethanol doses, consistent with diminished alcohol responsivity. Our AIE model yielded adult phenotypes that mirror adolescent‐typical ethanol responding; notably, a recent rodent study reported that adolescent binge exposure increases adult ethanol intake and reduces intoxication sensitivity, aligning closely with our findings [36]. Together, these data suggest that adolescent binge drinking confers lasting alcohol tolerance, perhaps through a HMGB1 neuroimmune mechanism that may underlie the increased risk of AUD development associated with an adolescent age of drinking onset.

Emerging studies have identified neuroimmune activation as contributing to increases in alcohol drinking [28, 29] and alcohol‐induced neuropathology [11, 37]. LPS (endotoxin) increases circulating plasma and brain levels of HMGB1 [38] and increases ethanol self‐administration in rodents [39]. Studies in AIE‐treated adult rats as well as in the post mortem human brain of individuals with AUD and an adolescent age of drinking onset find increased expression of neuroimmune signalling molecules, including HMGB1, throughout the brain [23, 26, 37]. HMGB1 binds to and activates TLR4, RAGE and other receptors, leading to nuclear translocation of NF‐κBp65, thereby contributing to complex proinflammatory signalling [28, 40]. Work from the Deak and Guerri groups demonstrated that adolescent alcohol exposure similarly engages neuroimmune pathways—altering cytokine/HPA dynamics and activating TLR4‐dependent signalling that contributes to microglial/astroglial activation, myelin disruption and lasting behavioural change [41, 42, 43, 44]. Acute binge drinking and chronic alcohol abuse increase intestinal permeability, allowing gut bacterial endotoxin components (e.g., LPS) to enter systemic circulation, inducing proinflammatory signals that reach the CNS [45], thereby altering cognition and drinking behaviours [46]. Our discovery that adolescent LPS mimics AIE‐induced low ethanol responsivity and decreases plasma HMGB1 levels across cumulative ethanol doses in adulthood is consistent with inflammation contributing to the development of alcohol tolerance.

Glycyrrhizic acid, a constituent of *Glycyrrhiza glabra* (licorice) classified by the FDA as generally recognized as safe as a food additive, is a selective HMGB1 inhibitor that binds to a pocket on HMGB1 blocking its proinflammatory activities through inactivation of released HMGB1 [35]. We report post‐AIE administration of glycyrrhizic acid restores adult alcohol responsivity to CON levels, blunts the persistent baseline induction of plasma HMGB1 and reverses HMGB1‐mediated neuroinflammation in the primary motor cortex despite near‐identical BECs compared to CONs at the time of testing. Emerging studies suggest that AIE and HMGB1‐mediated neuroinflammation can elicit chromatin remodelling in brain through epigenetic modifications, leading to changes in gene expression that contribute to alcohol‐induced neuropathology that are reversible [23, 34]. Reductions in acute alcohol responsivity across the cumulative ethanol challenge for different measures are likely dependent upon different brain regions but may involve epigenetic alterations in neurocircuitry that are reversible. For instance, inhibition of histone deacetylases (HDACs) reverses development of rapid tolerance to the anxiolytic effects of alcohol [47]. Furthermore, alcohol drinking in alcohol‐preferring rats has been linked to increases in HDAC activity [48]. While the brain regional contributions to AIE‐induced ethanol tolerance are likely related to individual ERB measures and remain to be fully elucidated, a recent study in adult mice found ethanol‐induced rotarod impairments related to cerebellar ethanol metabolism‐induced elevations of GABA levels [49]. Our data suggest that AIE‐induced developmental acquisition of alcohol tolerance in adulthood is reversible through blockade of HMGB1, providing a potential novel target for the development of therapeutics.

A methodological limitation of this study is that adult ERB assessments were performed at three different postnatal ages across experiments (P75, P80 and P95). Our primary inferences derive from within‐experiment comparisons conducted in age‐homogeneous cohorts under identical procedures, with comparable vehicle baselines and BECs, thereby limiting confounding by age in the statistical tests underlying each result. Notably, these ages lie within the young adult period in rats—a developmental window in which ethanol responsivity is comparatively stable relative to the pronounced shifts observed during adolescence (e.g., [50]). Nevertheless, we cannot exclude subtle age‐within‐adulthood effects; future studies will age‐match all cohorts and/or include postnatal day as a factor to directly evaluate potential age × treatment interactions on ERB outcomes.

In summary, we report AIE confers long‐lasting alcohol tolerance in adulthood across a range of cumulative ethanol doses as well as blunted ethanol‐induced HMGB1 plasma levels, consistent with neuroimmune tolerance. Adolescent LPS treatment, which models AIE‐induced HMGB1‐mediated neuroinflammation, confers lasting adult alcohol tolerance and blunted HMGB1 release across cumulative ethanol doses on the ERB, directly associating neuroinflammation with developmental acquisition of alcohol tolerance. Administration of the selective HMGB1 inhibitor glycyrrhizic acid reversed the AIE‐induced low ethanol responsivity in adult AIE‐treated animals, directly linking proinflammatory HMGB1 signalling to adolescent binge ethanol. These findings suggest a potential mechanistic target for the development of therapeutics for the treatment of AUD.

## Author Contributions

Study concept and design: Fulton T. Crews and Ryan P. Vetreno. Analysis and interpretation of data: Ryan P. Vetreno. Drafting of the manuscript: Ryan P. Vetreno. Critical revision of the manuscript for important intellectual content: Fulton T. Crews and Ryan P. Vetreno. Statistical analysis: Ryan P. Vetreno. Obtained funding: Fulton T. Crews and Ryan P. Vetreno. Study supervision: Fulton T. Crews and Ryan P. Vetreno. All the authors have made substantial contributions to the design of the work, the analysis and interpretation of the data, and the drafting of the work or its critical revision.

## Funding

Research reported in this publication was supported, in part, by the Foundation of Hope in Raleigh NC, the National Institute on Alcohol Abuse and Alcoholism (AA020024 (RPV/FTC), AA020023 (FTC) and AA019767 (FTC)) of the National Institutes of Health and the Bowles Center for Alcohol Studies.

## Disclosure

Dr. Vetreno reported no biomedical financial interests or potential conflicts of interest. Dr. Crews reported no biomedical financial interests or potential conflicts of interest.

## Conflicts of Interest

The authors declare no conflicts of interest. The content is solely the responsibility of the authors and does not necessarily represent the official views of the National Institutes of Health.

## Supporting information


**Figure S1:** adb_70119‐sup‐0001‐SupplementalInformation.pdf. **Baseline measures during ethanol response battery (ERB) assessment for Experiment 1. (A)** Baseline body weights of CON‐ (268 g ± 7.0) and AIE‐treated (265 g ± 5.0) animals at the time of ERB testing (i.e., P75). **(B)** Baseline body temperatures in CON‐ (38.5°C ± 0.17) and AIE‐treated animals (37.6°C ± 0.16) at the time of ERB testing. **(C)** Baseline time (s) on the accelerating rotarod between CON‐ (179.3 s ± 0.44) and AIE‐treated (179.2 s ± 0.63) animals. Dashed line indicates 3 min trial duration. **(D)** Baseline angle of slide on the tilting plant between CON‐ (76.7°C ± 0.68) and AIE‐treated (73.4°C ± 0.65) animals. *n* = 8 subjects/condition. Data are presented as mean ±SEM.
**Figure S2: Baseline measures during ethanol response battery (ERB) assessment for Experiment 2. (A)** Baseline body weights of CON‐ (275 g ± 12.3) and LPS‐treated (287 g ± 7.0) animals at the time of ERB testing (i.e., P80). **(B)** Baseline body temperatures in CON‐ (38.9°C ± 0.10) and LPS‐treated animals (38.4°C ± 0.12) at the time of ERB testing. **(C)** Baseline time (s) on the accelerating rotarod between CON‐ (179.6 s ± 0.38) and LPS‐treated (169.3 s ± 5.0) animals. Dashed line indicates 3 min trial duration. **(D)** Baseline angle of slide on the tilting plant between CON‐ (75.9°C ± 0.69) and AIE‐treated (77.4°C ± 0.66) animals. *n* = 8 subjects/condition. Data are presented as mean ±SEM.
**Figure S3: Baseline measures during ethanol response battery (ERB) assessment for Experiment 3. (A)** While female Wistar rats evidenced dramatic body weight gains across Experiment 3, neither AIE nor glycyrrhizic acid (glyz acid) treatment affected body weights. **(B)** Baseline body temperatures in CON‐ (vehicle: 37.7°C; glyz acid: 37.7°C) and AIE‐treated animals (vehicle: 37.6°C; glyz acid: 37.7°C) at the time of ERB testing (i.e., P9). **(C)** Baseline angle of slide on the tilting plant between CON‐ (vehicle: 81.7°; glyz acid: 79.3°) and AIE‐treated (vehicle: 77.4°; glyz acid: 78.6°) animals. *n* = 8 subjects/condition. Data are presented as mean ±SEM.

## Data Availability

The data that support the findings in this study are available from the corresponding author upon reasonable request.

## References

[1] S. K. Elvig , M. A. McGinn , C. Smith , M. A. Arends , G. F. Koob , and L. F. Vendruscolo , “Tolerance to Alcohol: A Critical yet Understudied Factor in Alcohol Addiction,” Pharmacology, Biochemistry and Behavior 204 (2021): 173155. 33631255 10.1016/j.pbb.2021.173155 PMC8917511

[2] B. Tabakoff and P. L. Hoffman , “Tolerance and the Etiology of Alcoholism: Hypothesis and Mechanism,” Alcoholism, Clinical and Experimental Research 12, no. 1 (1988): 184–186. 2964796 10.1111/j.1530-0277.1988.tb00157.x

[3] M. A. Schuckit , S. C. Risch , and E. O. Gold , “Alcohol Consumption, ACTH Level, and Family History of Alcoholism,” American Journal of Psychiatry 145, no. 11 (1988): 1391–1395. 2847567 10.1176/ajp.145.11.1391

[4] B. F. Grant , F. S. Stinson , and T. C. Harford , “Age at Onset of Alcohol use and DSM‐IV Alcohol Abuse and Dependence: A 12‐Year Follow‐Up,” Journal of Substance Abuse 13, no. 4 (2001): 493–504. 11775078 10.1016/s0899-3289(01)00096-7

[5] D. J. DeWit , E. M. Adlaf , D. R. Offord , and A. C. Ogborne , “Age at First Alcohol Use: A Risk Factor for the Development of Alcohol Disorders,” American Journal of Psychiatry 157, no. 5 (2000): 745–750. 10784467 10.1176/appi.ajp.157.5.745

[6] J. Heron , J. Macleod , M. R. Munafo , et al., “Patterns of Alcohol Use in Early Adolescence Predict Problem Use at Age 16,” Alcohol and Alcoholism 47, no. 2 (2012): 169–177. 22215001 10.1093/alcalc/agr156 PMC3284685

[7] L. A. Gardner , E. Stockings , K. E. Champion , M. Mather , and N. C. Newton , “Alcohol Initiation Before Age 15 Predicts Earlier Hazardous Drinking: A Survival Analysis of a 7‐Year Prospective Longitudinal Cohort of Australian Adolescents,” Addiction 119, no. 3 (2024): 518–529. 37926434 10.1111/add.16376

[8] L. Sjodin , J. Raninen , and P. Larm , “Early Drinking Onset and Subsequent Alcohol Use in Late Adolescence: A Longitudinal Study of Drinking Patterns,” Journal of Adolescent Health 74, no. 6 (2024): 1225–1230. 10.1016/j.jadohealth.2024.02.014 38493398

[9] M. H. Meier , A. Caspi , R. Houts , et al., “Prospective Developmental Subtypes of Alcohol Dependence From Age 18 to 32 Years: Implications for Nosology, Etiology, and Intervention,” Development and Psychopathology 25, no. 3 (2013): 785–800. 23880392 10.1017/S0954579413000175 PMC3725643

[10] M. A. Schuckit , “Low Level of Response to Alcohol as a Predictor of Future Alcoholism,” American Journal of Psychiatry 151, no. 2 (1994): 184–189. 8296886 10.1176/ajp.151.2.184

[11] F. T. Crews , D. L. Robinson , L. J. Chandler , et al., “Mechanisms of Persistent Neurobiological Changes Following Adolescent Alcohol Exposure: NADIA Consortium Findings,” Alcoholism, Clinical and Experimental Research 43, no. 9 (2019): 1806–1822. 31335972 10.1111/acer.14154 PMC6758927

[12] S. A. Brown , T. Brumback , K. Tomlinson , et al., “The National Consortium on Alcohol and NeuroDevelopment in Adolescence (NCANDA): A Multisite Study of Adolescent Development and Substance use,” Journal of Studies on Alcohol and Drugs 76, no. 6 (2015): 895–908. 26562597 10.15288/jsad.2015.76.895 PMC4712659

[13] J. C. Crabbe , R. L. Bell , and C. L. Ehlers , “Human and Laboratory Rodent Low Response to Alcohol: Is Better Consilience Possible?,” Addiction Biology 15, no. 2 (2010): 125–144. 20148776 10.1111/j.1369-1600.2009.00191.x PMC2853481

[14] R. P. Vetreno , J. Campbell , and F. T. Crews , “A Multicomponent Ethanol Response Battery Across a Cumulative Dose Ethanol Challenge Reveals Diminished Adolescent Rat Ethanol Responsivity Relative to Adults,” Advances in Drug and Alcohol Research 3 (2023): 11888. 38389807 10.3389/adar.2023.11888 PMC10880770

[15] A. M. White , J. G. Bae , M. C. Truesdale , S. Ahmad , W. A. Wilson , and H. S. Swartzwelder , “Chronic‐Intermittent Ethanol Exposure During Adolescence Prevents Normal Developmental Changes in Sensitivity to Ethanol‐Induced Motor Impairments,” Alcoholism, Clinical and Experimental Research 26, no. 7 (2002): 960–968. 12170104 10.1097/01.ALC.0000021334.47130.F9

[16] M. R. Watson , K. James , G. Mittleman , and D. B. Matthews , “Impact of Acute Ethanol Exposure on Body Temperatures in Aged, Adult and Adolescent Male Rats,” Alcohol 82 (2020): 81–89. 31408671 10.1016/j.alcohol.2019.08.001

[17] B. Lees , L. R. Meredith , A. E. Kirkland , B. E. Bryant , and L. M. Squeglia , “Effect of Alcohol Use on the Adolescent Brain and Behavior,” Pharmacology, Biochemistry, and Behavior 192 (2020): 172906. 32179028 10.1016/j.pbb.2020.172906 PMC7183385

[18] E. I. Varlinskaya and L. P. Spear , “Sensitization to Social Anxiolytic Effects of Ethanol in Adolescent and Adult Sprague‐Dawley Rats After Repeated Ethanol Exposure,” Alcohol 44, no. 1 (2010): 99–110. 20113878 10.1016/j.alcohol.2009.09.036 PMC2900816

[19] L. P. Spear , “Adolescents and Alcohol: Acute Sensitivities, Enhanced Intake, and Later Consequences,” Neurotoxicology and Teratology 41 (2014): 51–59. 24291291 10.1016/j.ntt.2013.11.006 PMC3943972

[20] R. M. Pautassi , M. E. Nizhnikov , N. E. Spear , and J. C. Molina , “Prenatal Ethanol Exposure Leads to Greater Ethanol‐Induced Appetitive Reinforcement,” Alcohol 46, no. 6 (2012): 585–593. 22698870 10.1016/j.alcohol.2012.05.004 PMC3532510

[21] W. Liu and F. T. Crews , “Adolescent Intermittent Ethanol Exposure Enhances Ethanol Activation of the Nucleus Accumbens While Blunting the Prefrontal Cortex Responses in Adult rat,” Neuroscience 293 (2015): 92–108. 25727639 10.1016/j.neuroscience.2015.02.014 PMC4821202

[22] M. Roberto and F. P. Varodayan , “Synaptic Targets: Chronic Alcohol Actions,” Neuropharmacology 122 (2017): 85–99. 28108359 10.1016/j.neuropharm.2017.01.013 PMC5479718

[23] F. T. Crews , V. Macht , and R. P. Vetreno , “Epigenetic Regulation of Microglia and Neurons by Proinflammatory Signaling Following Adolescent Intermittent Ethanol (AIE) Exposure and in Human AUD,” Advances in Drug and Alcohol Research 4 (2024): 12094. 38524847 10.3389/adar.2024.12094 PMC10957664

[24] M. El Gazzar , B. K. Yoza , X. Chen , B. A. Garcia , N. L. Young , and C. E. McCall , “Chromatin‐Specific Remodeling by HMGB1 and Linker Histone H1 Silences Proinflammatory Genes During Endotoxin Tolerance,” Molecular and Cellular Biology 29, no. 7 (2009): 1959–1971. 19158276 10.1128/MCB.01862-08 PMC2655606

[25] H. Yang , H. Wang , and U. Andersson , “Targeting Inflammation Driven by HMGB1,” Frontiers in Immunology 11 (2020): 484. 32265930 10.3389/fimmu.2020.00484 PMC7099994

[26] R. P. Vetreno and F. T. Crews , “Adolescent Binge Drinking Increases Expression of the Danger Signal Receptor Agonist HMGB1 and Toll‐Like Receptors in the Adult Prefrontal Cortex,” Neuroscience 226 (2012): 475–488. 22986167 10.1016/j.neuroscience.2012.08.046 PMC3740555

[27] M. Roberto , R. R. Patel , and M. Bajo , “Ethanol and Cytokines in the Central Nervous System,” Handbook of Experimental Pharmacology 248 (2018): 397–431. 29236160 10.1007/164_2017_77 PMC7886012

[28] J. Mayfield , L. Ferguson , and R. A. Harris , “Neuroimmune Signaling: A key Component of Alcohol Abuse,” Current Opinion in Neurobiology 23 (2013): 513–520. 23434064 10.1016/j.conb.2013.01.024 PMC3694992

[29] E. K. Grantham , R. Barchiesi , N. A. Salem , and R. D. Mayfield , “Neuroimmune Pathways as Targets to Reduce Alcohol Consumption,” Pharmacology, Biochemistry, and Behavior 222 (2023): 173491. 36400266 10.1016/j.pbb.2022.173491 PMC9906983

[30] L. R. Meredith , E. M. Burnette , E. N. Grodin , M. R. Irwin , and L. A. Ray , “Immune Treatments for Alcohol Use Disorder: A Translational Framework,” Brain, Behavior, and Immunity 97 (2021): 349–364. 34343618 10.1016/j.bbi.2021.07.023 PMC9044974

[31] R. P. Vetreno , J. P. Bohnsack , H. Kusumo , W. Liu , S. C. Pandey , and F. T. Crews , “Neuroimmune and Epigenetic Involvement in Adolescent Binge Ethanol‐Induced Loss of Basal Forebrain Cholinergic Neurons: Restoration With Voluntary Exercise,” Addiction Biology 25, no. 2 (2020): e12731. 30779268 10.1111/adb.12731 PMC6698434

[32] G. Paxinos and C. Watson , The rat Brain in Stereotaxic Coordinates (Academic Press, 2014). 10.1016/0165-0270(80)90021-7 6110810

[33] F. T. Crews , K. Nixon , and M. E. Wilkie , “Exercise Reverses Ethanol Inhibition of Neural Stem Cell Proliferation,” Alcohol 33, no. 1 (2004): 63–71. 15353174 10.1016/j.alcohol.2004.04.005

[34] F. T. Crews and R. P. Vetreno , “Cholinergic REST‐G9a Gene Repression Through HMGB1‐TLR4 Neuroimmune Signaling Regulates Basal Forebrain Cholinergic Neuron Phenotype,” Frontiers in Molecular Neuroscience 15 (2022): 992627. 36072299 10.3389/fnmol.2022.992627 PMC9441808

[35] L. Mollica , F. De Marchis , A. Spitaleri , et al., “Glycyrrhizin Binds to High‐Mobility Group Box 1 Protein and Inhibits Its Cytokine Activities,” Chemistry & Biology 14, no. 4 (2007): 431–441. 17462578 10.1016/j.chembiol.2007.03.007

[36] C. Ravasi , A. Salguero , L. Marengo , P. Penalver , and R. M. Pautassi , “Adolescent Binge Drinking in Male Wistar Rats Increases Ethanol Consumption and Reduces Intoxication Sensitivity in Early Adulthood Without Affecting Withdrawal,” American Journal of Drug and Alcohol Abuse 1‐12 (2025): 1–12. 10.1080/00952990.2025.2464644 39969851

[37] R. P. Vetreno , L. Qin , L. G. Coleman, Jr. , and F. T. Crews , “Increased Toll‐Like Receptor‐MyD88‐NFkappaB‐Proinflammatory Neuroimmune Signaling in the Orbitofrontal Cortex of Human Alcohol Use Disorder,” Alcoholism, Clinical and Experimental Research 45, no. 9 (2021): 1747–1761. 34415075 10.1111/acer.14669 PMC8526379

[38] V. Peek , L. M. Harden , J. Damm , et al., “LPS Primes Brain Responsiveness to High Mobility Group Box‐1 Protein,” Pharmaceuticals (Basel) 14, no. 6 (2021): 558. 34208101 10.3390/ph14060558 PMC8230749

[39] Y. A. Blednov , J. M. Benavidez , C. Geil , S. Perra , H. Morikawa , and R. A. Harris , “Activation of Inflammatory Signaling by Lipopolysaccharide Produces a Prolonged Increase of Voluntary Alcohol Intake in Mice,” Brain, Behavior, and Immunity 25, no. Suppl 1 (2011): S92–S105. 21266194 10.1016/j.bbi.2011.01.008 PMC3098320

[40] F. T. Crews , T. J. Walter , L. G. Coleman, Jr. , and R. P. Vetreno , “Toll‐Like Receptor Signaling and Stages of Addiction,” Psychopharmacology 234, no. 9–10 (2017): 1483–1498. 28210782 10.1007/s00213-017-4560-6 PMC5420377

[41] M. Pascual , A. Pla , J. Minarro , and C. Guerri , “Neuroimmune Activation and Myelin Changes in Adolescent Rats Exposed to High‐Dose Alcohol and Associated Cognitive Dysfunction: A Review With Reference to Human Adolescent Drinking,” Alcohol and Alcoholism 49, no. 2 (2014): 187–192. 24217958 10.1093/alcalc/agt164

[42] J. Montesinos , M. Pascual , M. Rodriguez‐Arias , J. Minarro , and C. Guerri , “Involvement of TLR4 in the Long‐Term Epigenetic Changes, Rewarding and Anxiety Effects Induced by Intermittent Ethanol Treatment in Adolescence,” Brain, Behavior, and Immunity 53 (2016): 159–171. 26686767 10.1016/j.bbi.2015.12.006

[43] F. Ibanez , J. Montesinos , J. R. Urena‐Peralta , C. Guerri , and M. Pascual , “TLR4 Participates in the Transmission of Ethanol‐Induced Neuroinflammation via Astrocyte‐Derived Extracellular Vesicles,” Journal of Neuroinflammation 16, no. 1 (2019): 136. 31272469 10.1186/s12974-019-1529-x PMC6610989

[44] T. L. Doremus‐Fitzwater and T. Deak , “Adolescent Neuroimmune Function and Its Interaction With Alcohol,” International Review of Neurobiology 161 (2022): 167–208. 34801169 10.1016/bs.irn.2021.08.006 PMC9204461

[45] S. Bala , M. Marcos , A. Gattu , D. Catalano , and G. Szabo , “Acute Binge Drinking Increases Serum Endotoxin and Bacterial DNA Levels in Healthy Individuals,” PLoS ONE 9, no. 5 (2014): e96864. 24828436 10.1371/journal.pone.0096864 PMC4020790

[46] S. Leclercq , P. de Timary , N. M. Delzenne , and P. Starkel , “The Link Between Inflammation, Bugs, the Intestine and the Brain in Alcohol Dependence,” Translational Psychiatry 7, no. 2 (2017): e1048. 28244981 10.1038/tp.2017.15 PMC5545644

[47] A. J. Sakharkar , H. Zhang , L. Tang , G. Shi , and S. C. Pandey , “Histone Deacetylases (HDAC)‐Induced Histone Modifications in the Amygdala: A Role in Rapid Tolerance to the Anxiolytic Effects of Ethanol,” Alcoholism, Clinical and Experimental Research 36, no. 1 (2012): 61–71. 21790673 10.1111/j.1530-0277.2011.01581.x PMC3208078

[48] S. Moonat , A. J. Sakharkar , H. Zhang , L. Tang , and S. C. Pandey , “Aberrant Histone deacetylase2‐Mediated Histone Modifications and Synaptic Plasticity in the Amygdala Predisposes to Anxiety and Alcoholism,” Biological Psychiatry 73, no. 8 (2013): 763–773. 23485013 10.1016/j.biopsych.2013.01.012 PMC3718567

[49] S. Jin , Q. Cao , F. Yang , et al., “Brain Ethanol Metabolism by Astrocytic ALDH2 Drives the Behavioural Effects of Ethanol Intoxication,” Nature Metabolism 3, no. 3 (2021): 337–351. 10.1038/s42255-021-00357-z PMC8294184 33758417

[50] L. P. Spear , “The Adolescent Brain and age‐Related Behavioral Manifestations,” Neuroscience and Biobehavioral Reviews 24, no. 4 (2000): 417–463. 10817843 10.1016/s0149-7634(00)00014-2

