# Supplementary material for: Adolescent Binge Ethanol Exposure Confers Lasting Adult Alcohol Tolerance due to Neuroimmune Activation: Reversal by Inhibition of HMGB1
Source: Addict Biol. 2026 Mar 4;31(3):e70119. doi: 10.1111/adb.70119 (PMC12959956; doi:10.1111/adb.70119)
Supplement: Supplementary file 1 — Figure S1: adb_70119‐sup‐0001‐SupplementalInformation.pdf. Baseline measures during ethanol response battery (ERB) assessment for Experiment 1. (A) Baseline body weights of CON‐ (268 g ± 7.0) and AIE‐treated (265 g ± 5.0) animals at the time of ERB testing (i.e., P75). (B) Baseline body temperatures in CON‐ (38.5°C ± 0.17) and AIE‐treated animals (37.6°C ± 0.16) at the time of ERB testing. (C) Baseline time (s) on the accelerating rotarod between CON‐ (179.3 s ± 0.44) and AIE‐treated (179.2 s ± 0.63) animals. Dashed line indicates 3 min trial duration. (D) Baseline angle of slide on the tilting plant between CON‐ (76.7°C ± 0.68) and AIE‐treated (73.4°C ± 0.65) animals. n = 8 subjects/condition. Data are presented as mean ±SEM. Figure S2: Baseline measures during ethanol response battery (ERB) assessment for Experiment 2. (A) Baseline body weights of CON‐ (275 g ± 12.3) and LPS‐treated (287 g ± 7.0) animals at the time of ERB testing (i.e., P80). (B) Baseline body temperatures in CON‐ (38.9°C ± 0.10) and LPS‐treated animals (38.4°C ± 0.12) at the time of ERB testing. (C) Baseline time (s) on the accelerating rotarod between CON‐ (179.6 s ± 0.38) and LPS‐treated (169.3 s ± 5.0) animals. Dashed line indicates 3 min trial duration. (D) Baseline angle of slide on the tilting plant between CON‐ (75.9°C ± 0.69) and AIE‐treated (77.4°C ± 0.66) animals. n = 8 subjects/condition. Data are presented as mean ±SEM. Figure S3: Baseline measures during ethanol response battery (ERB) assessment for Experiment 3. (A) While female Wistar rats evidenced dramatic body weight gains across Experiment 3, neither AIE nor glycyrrhizic acid (glyz acid) treatment affected body weights. (B) Baseline body temperatures in CON‐ (vehicle: 37.7°C; glyz acid: 37.7°C) and AIE‐treated animals (vehicle: 37.6°C; glyz acid: 37.7°C) at the time of ERB testing (i.e., P9). (C) Baseline angle of slide on the tilting plant between CON‐ (vehicle: 81.7°; glyz acid: 79.3°) and AIE‐treated (vehicle: 77.4°; glyz a [file ADB-31-e70119-s001.pdf]

Adolescent Binge Ethanol Exposure Confers Lasting Adult Alcohol Tolerance due to Neuroimmune Activation: Reversal by Inhibition of HMGB1

Supplemental Information

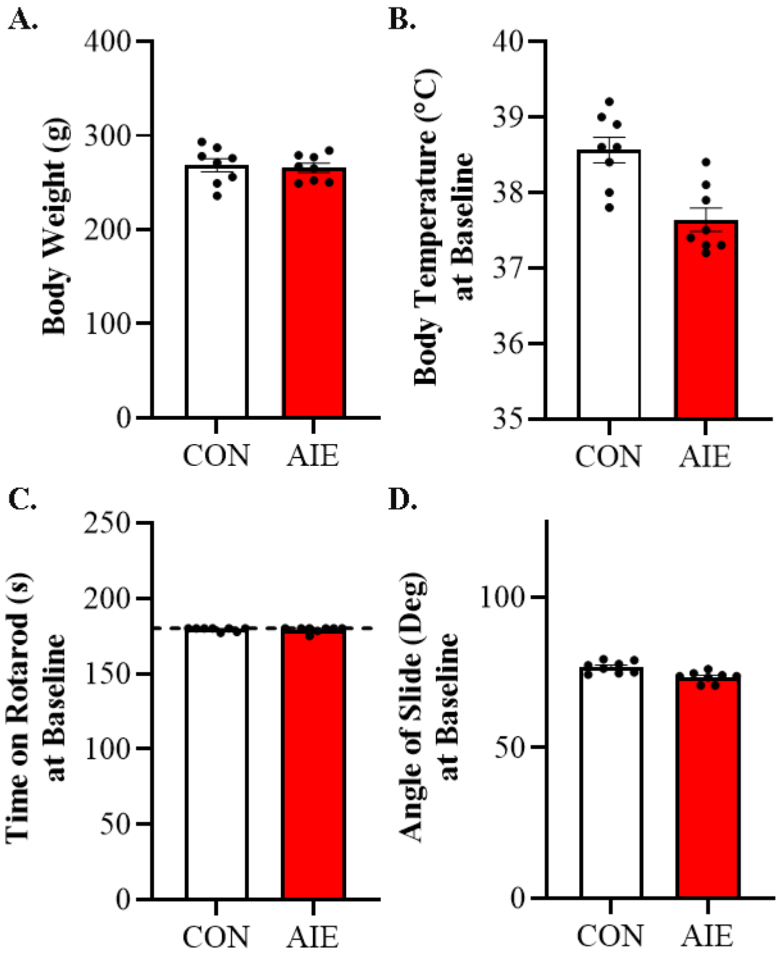

Figure S1. Baseline measures during ethanol response battery (ERB) assessment for Experiment 1. (A) Baseline body weights of CON- (268 g ±7.0) and AIE-treated (265 g ±5.0) animals at the time of ERB testing (i.e., P75). **(B)** Baseline body temperatures in CON- (38.5°C ±0.17) and AIE-treated animals (37.6°C ±0.16) at the time of ERB testing. **(C)** Baseline time (s) on the accelerating rotarod between CON- (179.3 s ±0.44) and AIE-treated (179.2 s ±0.63) animals. Dashed line indicates 3 min trial duration. **(D)** Baseline angle of slide on the tilting plant between CON- (76.7° ±0.68) and AIE-treated (73.4° ±0.65) animals. n=8 subjects/condition. Data presented as mean ±SEM.

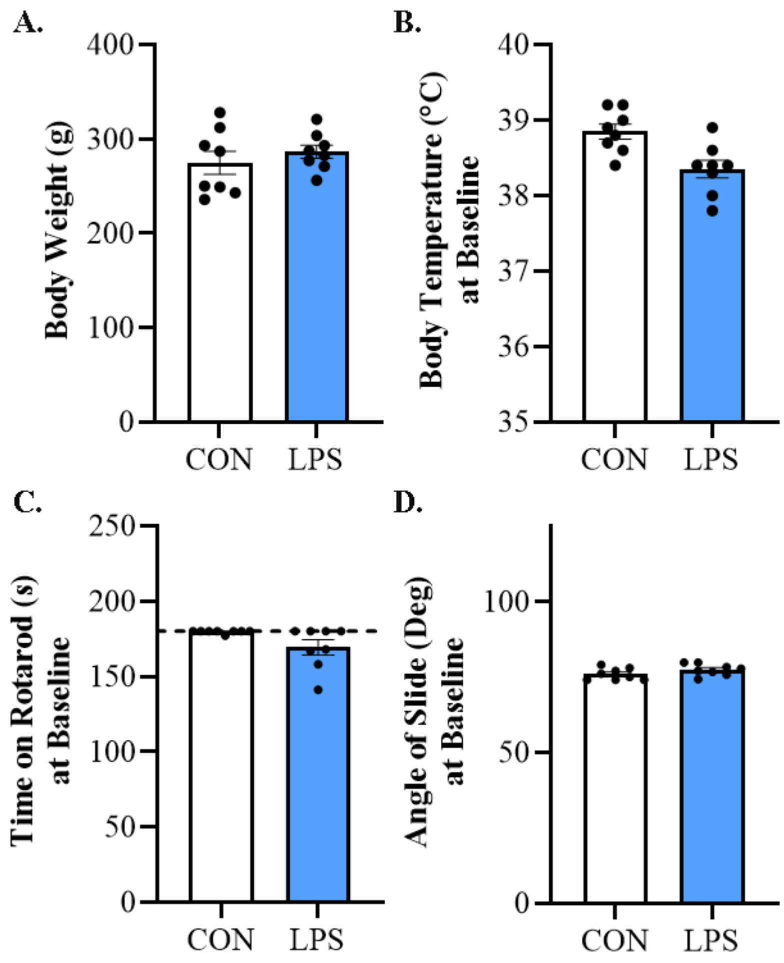

Figure S2. Baseline measures during ethanol response battery (ERB) assessment for Experiment 2. (A) Baseline body weights of CON- (275 g ±12.3) and LPS-treated (287 g ±7.0) animals at the time of ERB testing (i.e., P80). **(B)** Baseline body temperatures in CON- (38.9°C ±0.10) and LPS-treated animals (38.4°C ±0.12) at the time of ERB testing. **(C)** Baseline time (s) on the accelerating rotarod between CON- (179.6 s ±0.38) and LPS-treated (169.3 s ±5.0) animals. Dashed line indicates 3 min trial duration. **(D)** Baseline angle of slide on the tilting plant between CON- (75.9° ±0.69) and AIE-treated (77.4° ±0.66) animals. n=8 subjects/condition. Data presented as mean ±SEM.

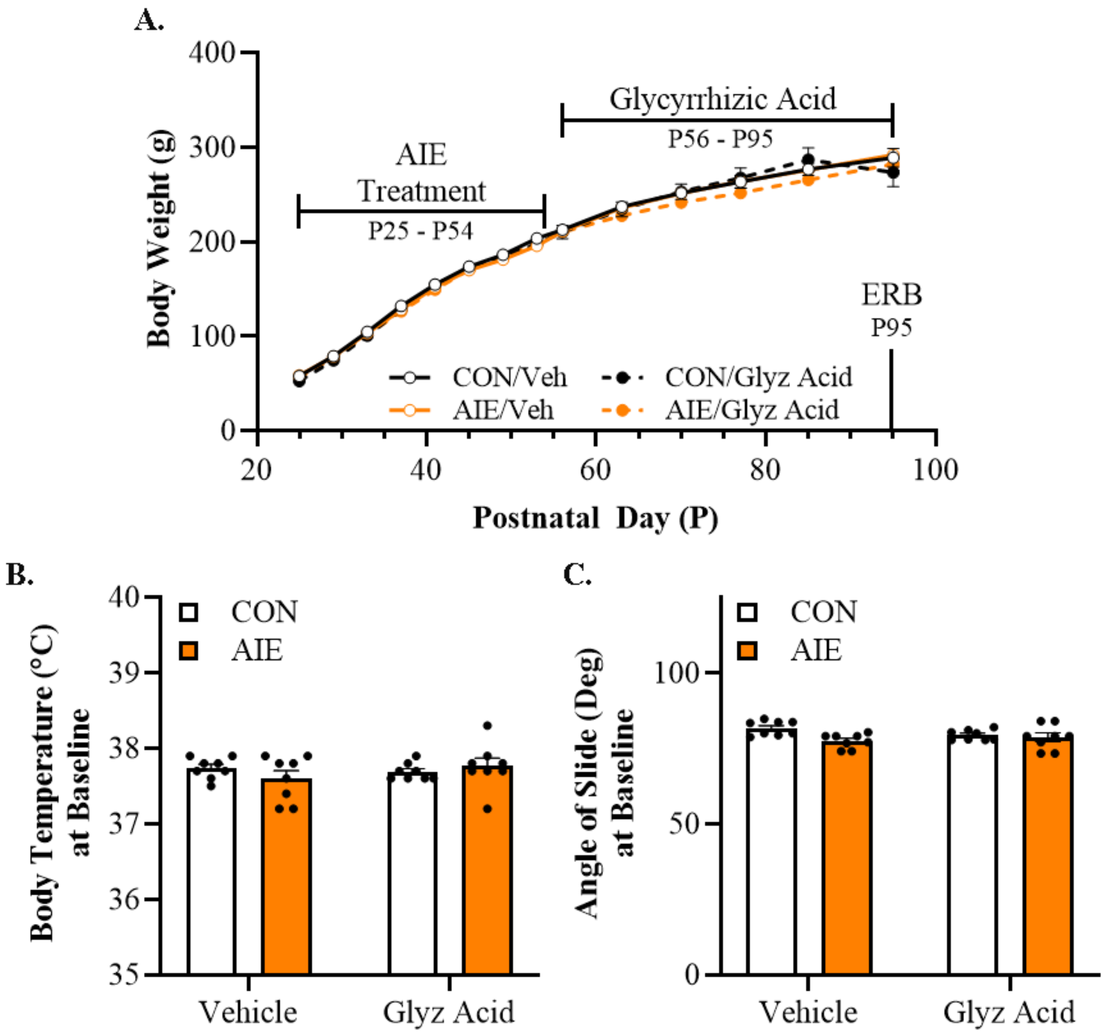

Figure S3. Baseline measures during ethanol response battery (ERB) assessment for Experiment 3. (A) While female Wistar rats evidenced dramatic body weight gains across Experiment 3, neither AIE or glycyrrhizic acid (glyz acid) treatment affected body weights. **(B)** Baseline body temperatures in CON- (Vehicle: 37.7°C; Glyz Acid: 37.7°C) and AIE-treated animals (Vehicle: 37.6°C; Glyz Acid: 37.7°C) at the time of ERB testing (i.e., P9). **(C)** Baseline angle of slide on the tilting plant between CON- (Vehicle: 81.7°; Glyz Acid: 79.3°) and AIE-treated (Vehicle: 77.4°; Glyz Acid: 78.6°) animals. n=8 subjects/condition. Data presented as mean ±SEM.
